# Fragment-based discovery of dual ligand pharmacophores for lipid-sensing transcription factors for designed polypharmacology†

**DOI:** 10.1039/d5md00531k

**Published:** 2025-07-23

**Authors:** Tanja Stiller, Silke Duensing-Kropp, Julian A. Marschner, Daniel Merk

**Affiliations:** a Department of Pharmacy, Ludwig-Maximilians-Universität München 81377 Munich Germany daniel.merk@cup.lmu.de

## Abstract

Designed polypharmacology aims to exploit additive or synergistic effects of simultaneous multi-target modulation. Multifactorial diseases like metabolic dysfunction requiring multi-drug treatment may significantly benefit from this concept. To identify multi-target lead pharmacophores for the development of designed dual ligands, we performed a focused two-stage screening of fatty acid mimetic fragments for modulation of the nuclear receptors THR, PPAR, FXR and RXR which are involved in transcriptional regulation of metabolic balance. Dual, multiple and pan-agonist hits were retrieved for various combinations of these targets of interest and preliminary SAR evaluation yielded dual agonist and pan-agonist fragments with attractive potency and efficacy as valuable leads for polypharmacology.

## Abstract 2

A two-stage focused fragment screening and preliminary structure activity relationship evaluation identified dual modulator fragments with attractive multi-target activity profiles as leads for designed polypharmacology.

## Graphical abstract

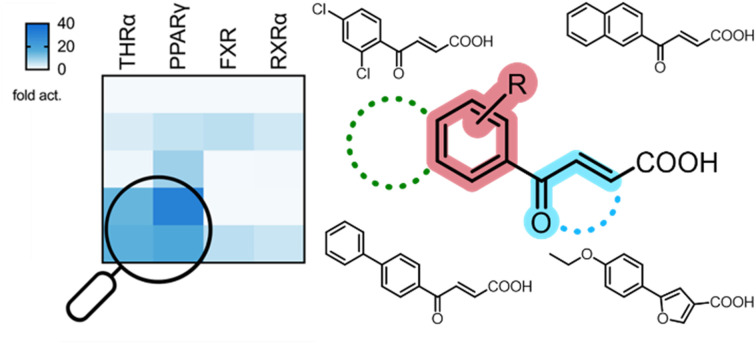

## Introduction

Designed polypharmacology is an innovative strategy in medicinal chemistry aiming to develop small molecule drugs that achieve enhanced therapeutic efficacy by simultaneous modulation of two or more targets.^1–3^ This is particularly attractive in multifactorial diseases like the metabolic syndrome (MetS) or chronic inflammation which involve the concomitant dysregulation of several metabolic pathways and signaling systems.^4–8^ The intentional modulation of two or more targets associated with a disease can have beneficial impact by several mechanisms such as reduced shunting effects, inhibition of redundant signaling pathways, and synergies from interference at several points of a biochemical cascade.^1,2,9^ Current treatment of multifactorial pathologies and multimorbidity is characterized by a heavy use of drug combinations^5,10,11^ underlining the potential of multi-target modulation by a single drug and the potential of designed polypharmacology to overcome multi-drug treatment (*i.e.*, polypharmacy).^1,2^

Metabolic dysfunction-associated steatohepatitis (MASH), formerly known as non-alcoholic steatohepatitis (NASH), is such a multifactorial condition that could benefit from designed polypharmacology. It is a severe hepatic manifestation of the MetS and has alarming prevalence.^12–14^ MASH is characterized by liver steatosis and inflammation leading to cell damage and hepatic fibrosis which can ultimately progress to liver cirrhosis and hepatocellular carcinoma.^12–14^ It thus presents as a severe health issue but available pharmacotherapy is still very limited and multiple approaches have failed in clinical development due to a lack of efficacy.^15^ Combination therapies are therefore getting into the focus of drug development for MASH^15,16^ highlighting designed polypharmacology as a potential avenue to drugs with improved efficacy in this indication.

Several approved or advanced experimental agents in MetS and MASH treatment like obeticholic acid, pioglitazone and resmetirom act as agonists of ligand-activated transcription factors (thyroid hormone receptor (THR), peroxisome proliferator-activated receptor (PPAR) γ, and farnesoid X receptor (FXR)), respectively.^17–19^ These nuclear receptors (NRs) regulate metabolic balance in different tissues and *via* different pathways. THR and FXR are key regulators of hepatic metabolism and lipid clearance.^18,20–22^ Additionally, FXR has a critical role in gut–liver-signaling and acts as liver protective transcription factor.^21–24^ PPARγ is the master regulator of adipose tissue homeostasis and involved in insulin-sensitivity and glucose balance.^25,26^ Therefore, simultaneous activation of two or more of these NRs may result in synergistic therapeutic effects and improved efficacy. Additionally, THR, PPARγ and FXR act as heterodimers with the retinoid X receptor (RXR)^27^ and synergies may also be achieved *via* simultaneous activation of both heterodimer partners.

**Fig. 1:**
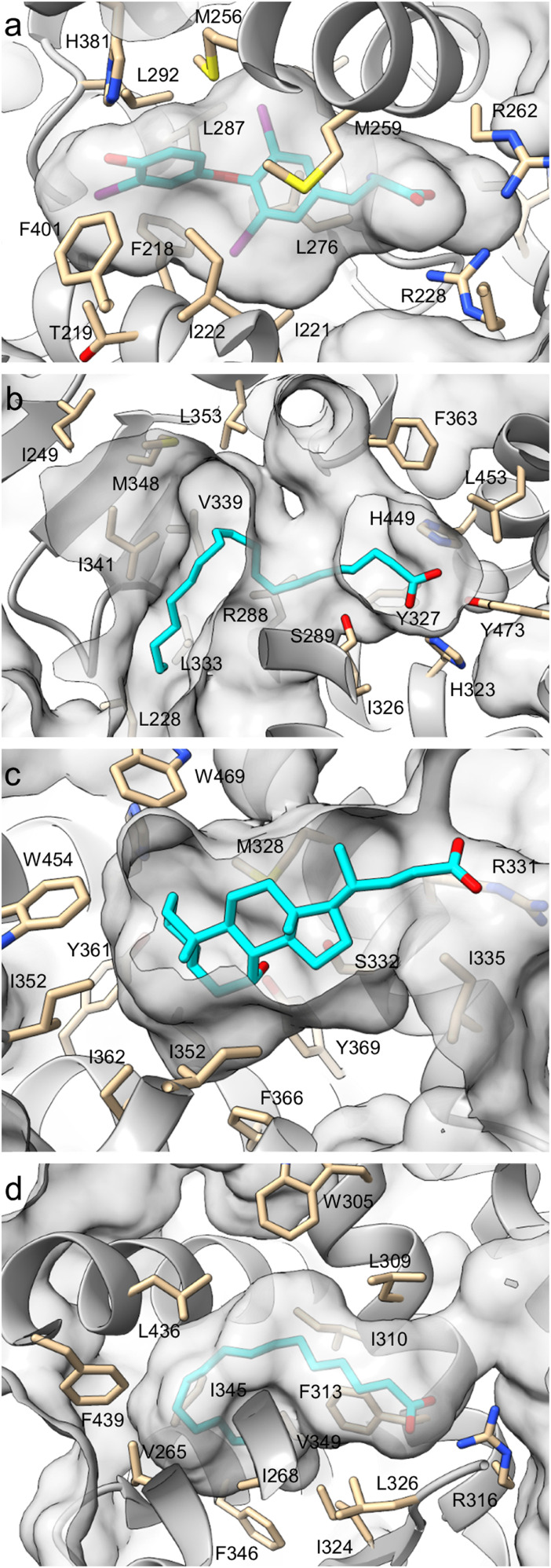
Ligand binding site comparison of THRα (a; pdb ID 2h79;^29^ ligand: T3), PPARγ (b; pdb ID 6mcz;^30^ ligand: arachidonic acid), FXR (c; pdb ID 6hl1;^31^ ligand: CDCA) and RXRα (d; pdb ID 7a77;^32^ ligand: palmitic acid). Ligand pockets were extracted from co-crystal structures of the targets of interest in complex with natural ligands. The structures were oriented with the natural ligands' polar end on the right. Selected residues lining the pocket are shown.

Based on these considerations, we sought to identify multitarget pharmacophores for THR, PPARγ, FXR and RXR in a fragment-based approach. These four targets of interest recognize fatty acids (PPARγ, RXRα) and other lipids (THRα, FXR) and their ligand binding sites albeit differing in size and shape share common characteristics (Fig. 1). The orthosteric pockets of all four receptors are generally hydrophobic but comprise a highly polar end enabling ionic interactions with Arg residues (THRα, FXR, RXRα) or extensive hydrogen bonding with Tyr, His and Ser residues (PPARγ). Therefore, we hypothesized potential of fatty acid mimetics^28^ as multi-target pharmacophores, and fragments bearing a carboxylic acid motif for strong polar contacts appeared suitable to scan for dual binders. In a two-stage focused screening of custom carboxylic acid containing fragment libraries, we obtained dual and multiple target hits for various combinations of THR, PPARγ, FXR and RXR and their preliminary SAR evaluation yielded valuable leads for polypharmacology.

## Results & discussion

To identify common pharmacophores of THR, PPARγ, FXR and RXR for the development of dual/multiple ligands, we followed a fragment-based approach. The four NRs of interest recognize fatty acids (PPAR, RXR) and other acidic lipids (THR, FXR) as natural ligands and agonist binding to these receptors typically involves salt bridges or strong H-bonds between an acidic motif of the ligand and basic residues of the receptor (Fig. 1). Therefore, we reasoned that fragments binding to two or more of the receptors of interest would be accessible from focused screening of carboxylic acid containing fragments.

**Fig. 2:**
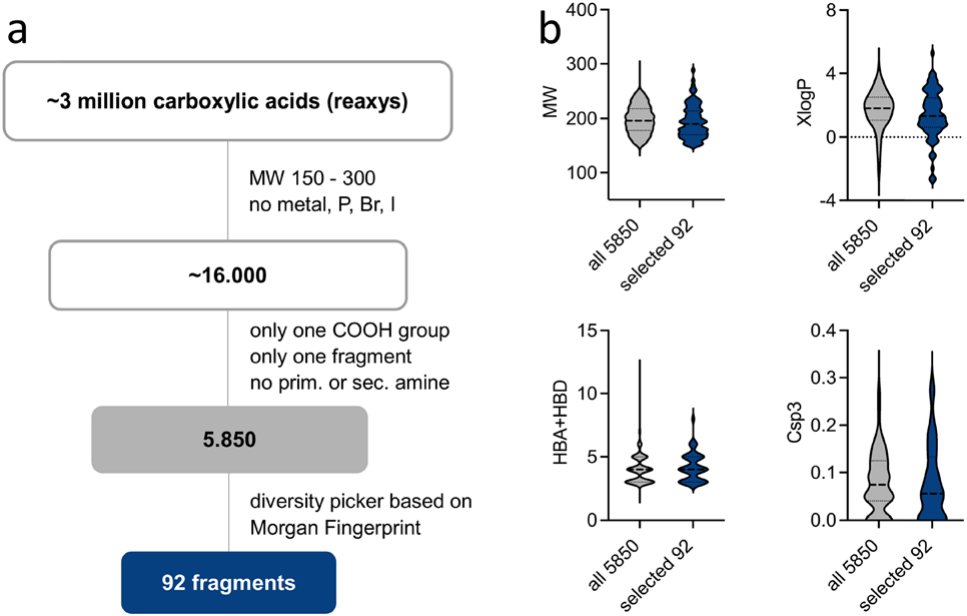
Compilation of the fragment screening set. (a) The flowchart illustrates the structure search and selection process for the fragment screening set. (b) The selected 92 fragments reflected the molecular weight (MW), the lipophilicity (XlogP), H-bond donor/acceptor (HBA + HBD) and sp3 character (Csp3) distribution of the 5850 available carboxylic acid containing fragments.

To assemble a focused fragment screening set, we identified 5850 commercially available carboxylic acid containing fragments (MW ≤ 300 g mol^−1^) and used a diversity picker based on Morgan fingerprints^33^ to select chemically diverse entities (Fig. 2a). With this procedure, we selected 92 carboxylic acids (70 unique scaffolds) with high diversity (mean ± SD Tanimoto similarity computed of Morgan fingerprints = 0.21 ± 0.06) and favorable fragment features (Fig. 2b).^34^ The 92 selected carboxylic acid fragments reflected the molecular feature distribution (MW, XlogP, HBA/HBD, Csp3) of the available carboxylic acid containing fragments and formed a representative screening set (Fig. 2b).

**Fig. 3:**
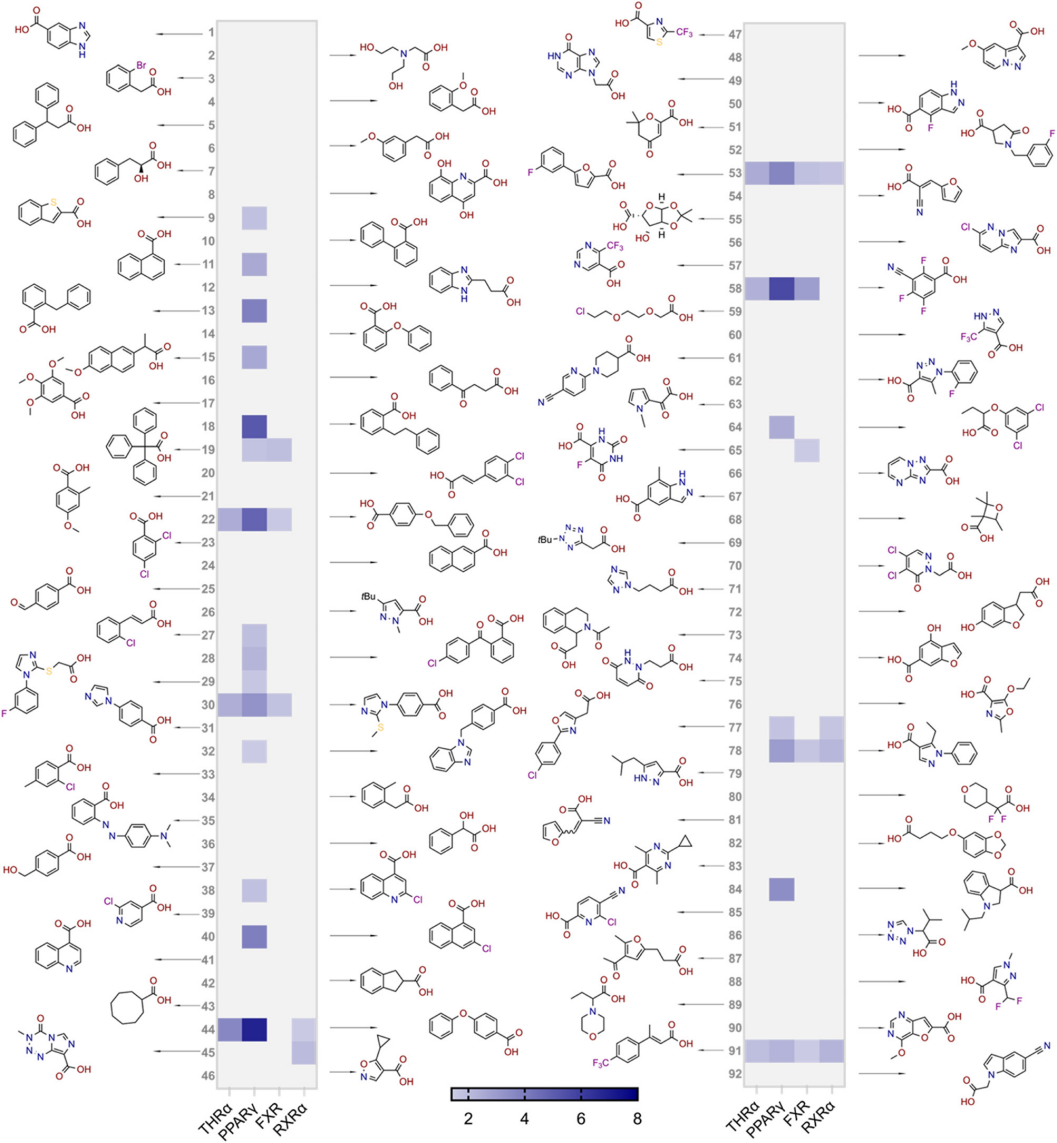
Results of the carboxylic acid fragment screening for modulation of THRα, PPARγ, FXR and RXRα. Compounds were tested at 100 μM in uniform Gal4-hybrid reporter gene assays. The heatmap shows the mean fold activation *vs.* DMSO (0.1%) treated cells; *n* = 3.

The focused fragment collection was then screened for activation of THRα, PPARγ, FXR and RXRα in uniform Gal4-hybrid reporter gene assays^35^ at 100 μM test concentration in three independent repeats (Fig. 3). A high hit-rate of 24 fragments caused statistically significant (*t*-test, *p* < 0.05) activation of at least one receptor of interest supporting the fragment-based approach.^36,37^ The highest number of actives was retrieved for PPARγ (22/92), followed by FXR (8/92), RXRα (6/92) and THRα (6/92). However, only eight fragments exhibited a multi-target profile on the targets of interest highlighting the challenge of identifying suitable lead pharmacophores for designed polypharmacology.^1^

**Fig. 4:**
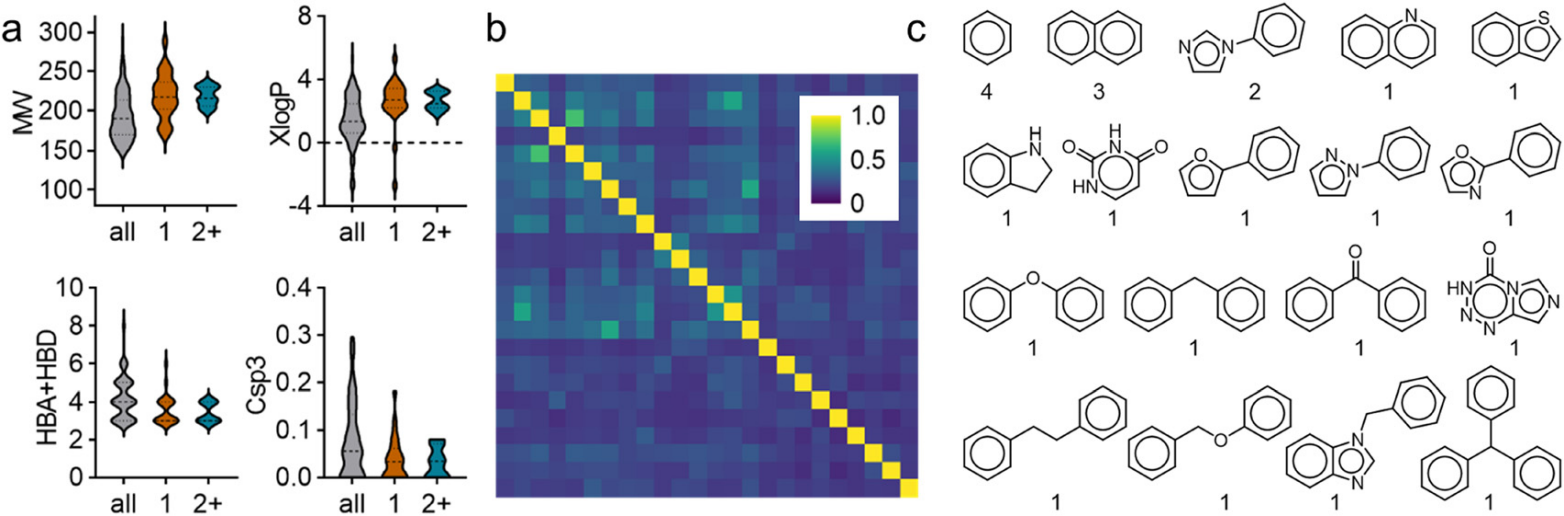
Molecular features of the fragment hits. (a) Fragment hits tended to have higher lipophilicity (XlogP), less H-bond donor/acceptor (HBA + HBD) features and less sp3 character (Csp3). (b) The scaffold hits retained high chemical diversity as illustrated by low pairwise Tanimoto similarity computed on Morgan fingerprints. (c) The 24 fragment hits contained 18 unique atomic scaffolds. Numbers indicate frequency of a scaffold in the hits.

**Fig. 5:**
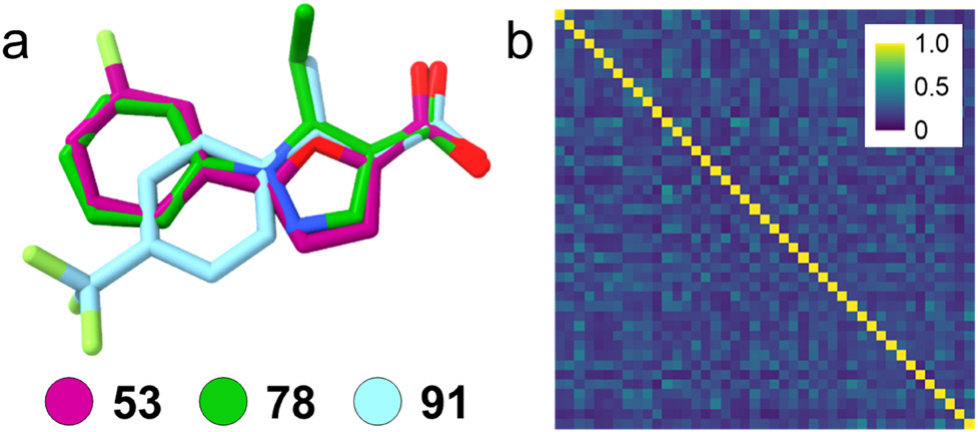
(a) The top hits 53, 78 and 91 aligned well with low RMSD (0.29–0.33) suggesting arylpropanoic/-butanoic acid as privileged multi-target scaffold for the targets of interest. Flexible alignment was performed with RDKit software. (b) Despite the common structural elements, the focused second-stage screening set was chemically diverse. The heatmap shows the pairwise Tanimoto similarity computed on Morgan fingerprints.

In comparative analysis of molecular properties (Fig. 4a), the hits tended to be more lipophilic (XlogP) and have less sp3 character (Csp3) and hydrogen bond donor/acceptor (HBA + HBD) features than the average of the screening set. These trends were even more pronounced for fragments hitting two or more targets. Nevertheless, the hits retained chemical diversity with low pairwise Tanimoto similarity computed on Morgan fingerprints^33^ (Fig. 4b) and high scaffold diversity (18 unique atomic scaffolds^38^ in 24 hits; Fig. 4c). Full dose–response profiling of the eight fragments hitting at least two targets of interest (Table 1) validated all hits except 19 and 58 as dual/multiple agonists with intermediate to high micromolar potencies. Despite higher rate of actives for PPARγ and THRα compared to FXR and RXRα, a lead fragment for every combination of the targets of interest was identified. Particularly 53, 78, and 91 emerged as promising fragment hits for designed polypharmacology with favorable multi-target activity on the NRs of interest. Comparative structural evaluation revealed that these hits aligned with low RMSD (Fig. 5a) suggesting the common arylpropanoic or arylbutanoic acid as privileged multi-target ligand skeleton.

**Table 1:** Activity of the multi-target fragment hits on the targets of interest

ID	Structure	EC_50_^a^ (max. fold activation)			
		THRα	PPARγ	FXR	RXRα
19	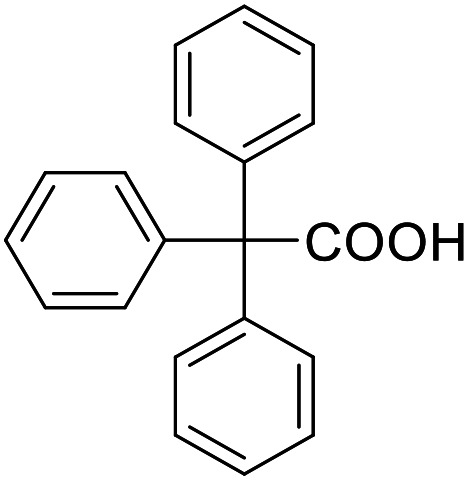	Inverse agonist	Inactive	Inactive	Inactive
		IC_50_ = 36 ± 4 μM	(1–300 μM)	(1–300 μM)	(1–300 μM)
		(41 ± 5% remain.)			
22	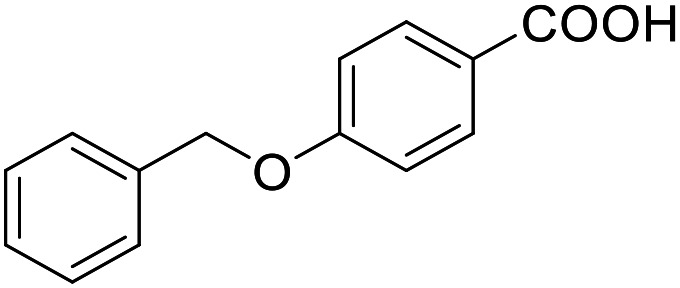	95 ± 9 μM	136 ± 19 μM	Inactive	Inactive
		(8.8 ± 0.5-fold)	(28 ± 2-fold)	(1–300 μM)	(1–300 μM)
30	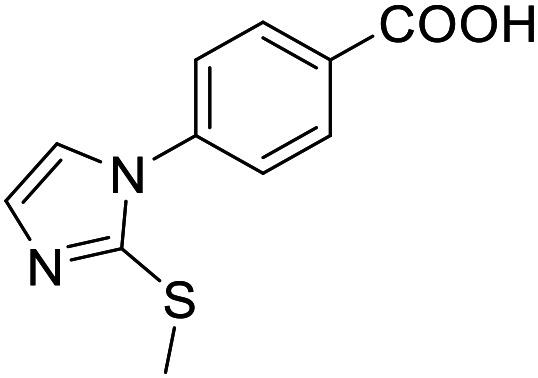	185 ± 16 μM	53 ± 5 μM	162 ± 31 μM	Inactive
		(2.5 ± 0.4-fold)	(2.4 ± 0.1-fold)	(2.2 ± 0.3-fold)	(1–300 μM)
44	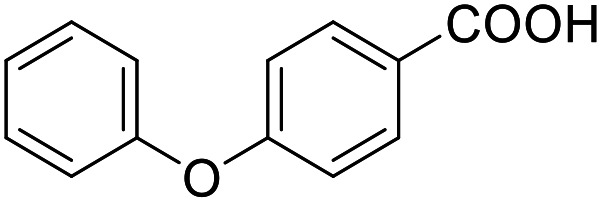	105 ± 28 μM	99 ± 12 μM	Inactive	Inactive
		(18 ± 3-fold)	(38 ± 3-fold)	(1–300 μM)	(1–300 μM)
53	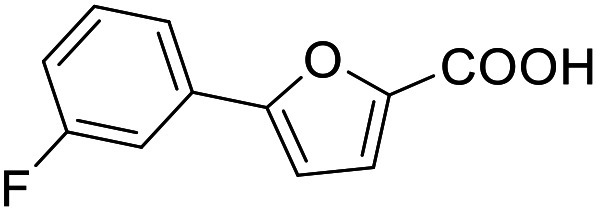	194 ± 54 μM	126 ± 17 μM	53 ± 10 μM	54 ± 9 μM
		(5 ± 1-fold)	(14 ± 1-fold)	(1.9 ± 0.1-fold)	(2.2 ± 0.13-fold)
58	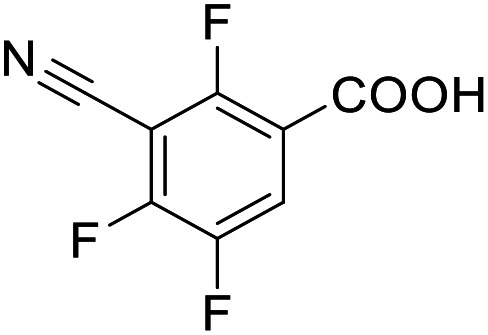	Inactive	39 ± 3 μM	Inactive	Inactive
		(1–300 μM)	(9.6 ± 0.5-fold)	(1–300 μM)	(1–300 μM)
78	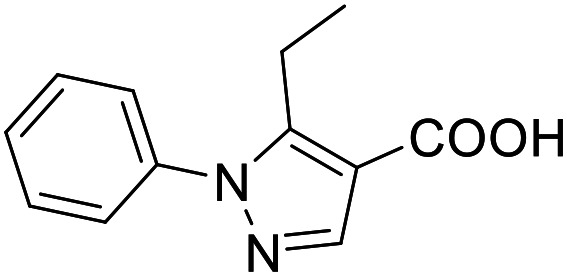	112 ± 44 μM	237 ± 37 μM	Inactive	166 ± 10 μM
		(2.0 ± 0.1-fold)	(6 ± 1-fold)	(1–300 μM)	(1.6 ± 0.1-fold)
91	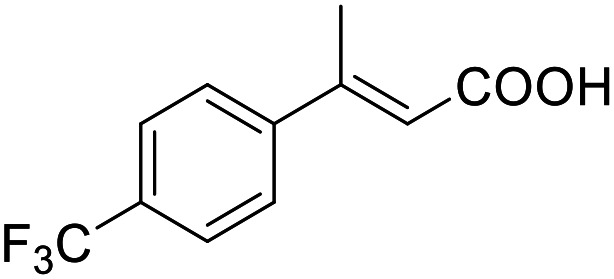	92 ± 29 μM	255 ± 15 μM	Inactive	134 ± 41 μM
		(2.7 ± 0.3-fold)	(4.3 ± 0.2-fold)	(1–300 μM)	(1.9 ± 0.9-fold)

a NR modulation was determined in uniform Gal4-hybrid reporter gene assays. Fold activation refers to the maximum reporter activation compared to DMSO (0.1%) treated cells. Data are the mean ± S.E.M.; *n* ≥ 3.

**Fig. 6:**
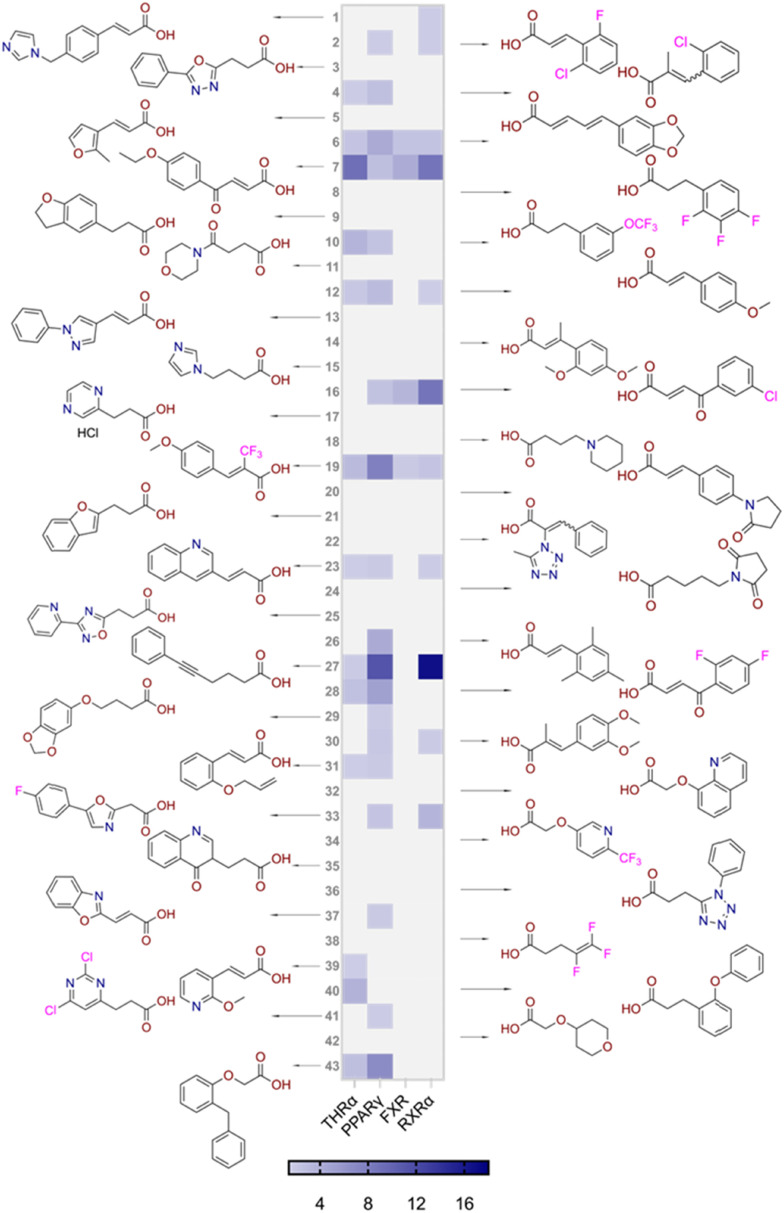
Results of the second-stage carboxylic acid fragment screening for modulation of THRα, PPARγ, FXR and RXRα. Compounds were tested at 100 μM in uniform Gal4-hybrid reporter gene assays. The heatmap shows the mean fold activation *vs.* DMSO (0.1%) treated cells; *n* = 3.

Based on this observation we assembled a focused second-stage fragment screening set of 43 propanoic acid, butyric acid and acrylic acid derivatives with lipophilic backbone (MW 207 ± 24; log *P* 1.8 ± 0.7; Fig. 6). Despite the common structural elements, the focused set was designed to retain chemical diversity (Fig. 5b).

Screening of the focused set for modulation of the targets of interest (Fig. 6) indeed resulted in substantially higher hit-rate with 22/43 fragments activating at least one of the studied NRs and 14/43 fragments exhibiting multi-target activity corroborating the focused set. PPARγ (18/43) remained the NR with the highest hit-rate but with less difference to THRα (13/43) and RXRα (11/43) than in the first round of screening. The hit-rate for FXR (4/43) was low indicating that the arylpropanoic/-butanoic acid scaffold might be less privileged for this receptor. The second stage screening results indicated that various carboxylic acid chains (propanoic acid, butanoic acid, acrylic acid, oxobutenoic acid) were tolerated but that a hydrophobic aromatic motif was required while more polar and aliphatic systems were not active. Additionally, linear fragments appeared favored over L-shaped geometries.

The second screening yielded the fragments 2.6, 2.7, 2.12, 2.16, 2.19, 2.27, 2.28, and 2.43 as further promising leads for designed polypharmacology and full profiling confirmed agonism on at least one target of interest (Table 2). Fragments 2.6, 2.7 and 2.19 activated all four receptors with intermediate to high micromolar potencies, 2.27 exhibited weak dual PPARγ/RXRα agonism and 2.28 emerged as dual THRα/PPARγ agonist. The arylpropanoic/-butanoic acid motif and the phenyloxobutenoic acid residue thus emerged from the second stage screening as promising fatty acid mimetic multi-target pharmacophores for further evaluation.

**Table 2:** Activity of the multi-target fragment hits on the targets of interest

ID	Structure	EC_50_^a^ (max. fold activation)			
		THRα	PPARγ	FXR	RXRα
2.6	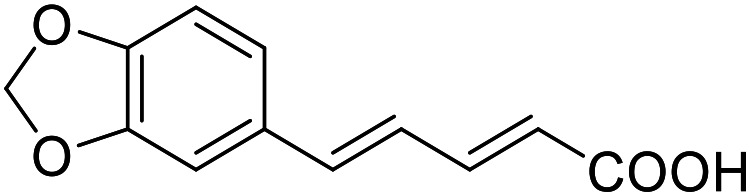	225 ± 91 μM	137 ± 8 μM	138 ± 44 μM	112 ± 19 μM
		(5.0 ± 1.4-fold)	(9.3 ± 0.5-fold)	(5.2 ± 0.8-fold)	(3.4 ± 0.2-fold)
2.7	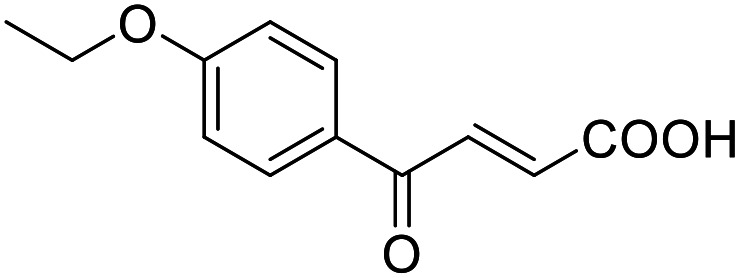	27 ± 1 μM	25.9 ± 0.4 μM	26 ± 1 μM	33 ± 6 μM
		(8.9 ± 0.5-fold)	(31.2 ± 0.6-fold)	(9.5 ± 0.4-fold)	(8 ± 1-fold)
2.12	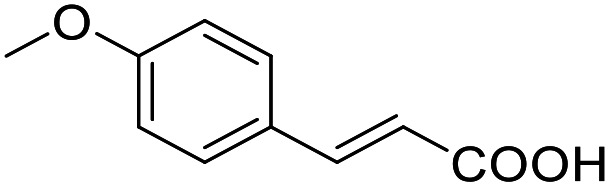	Inactive	126 ± 9 μM	Inactive	Inactive
		(1–300 μM)	(6.7 ± 0.3-fold)	(1–300 μM)	(1–300 μM)
2.16	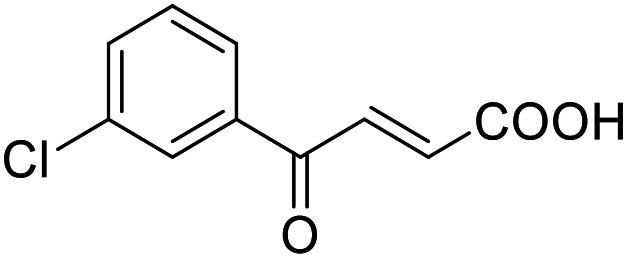	Inactive	34 ± 7 μM	Inactive	Inactive
		(1–300 μM)	(8.2 ± 0.5-fold)	(1–300 μM)	(1–300 μM)
2.19	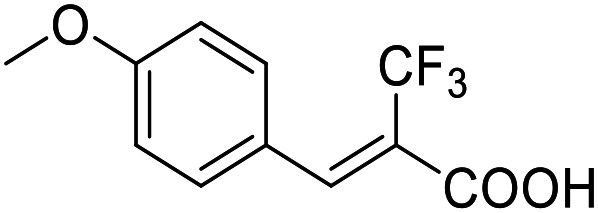	55 ± 19 μM	86 ± 8 μM	108 ± 22 μM	55 ± 8 μM
		(2.6 ± 0.3-fold)	(21 ± 1-fold)	(3.2 ± 0.4-fold)	(3.5 ± 0.1-fold)
2.27	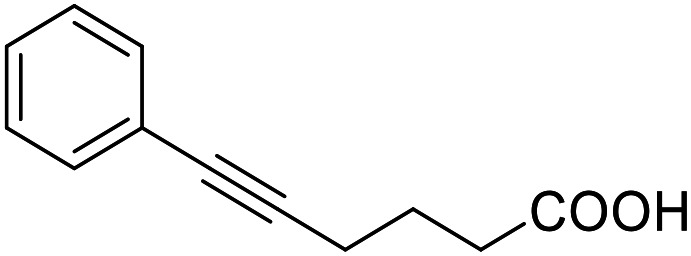	Inactive	106 ± 5 μM	Inactive	129 ± 11 μM
		(1–300 μM)	(25.0 ± 0.8-fold)	(1–300 μM)	(22.5 ± 1.5-fold)
2.28	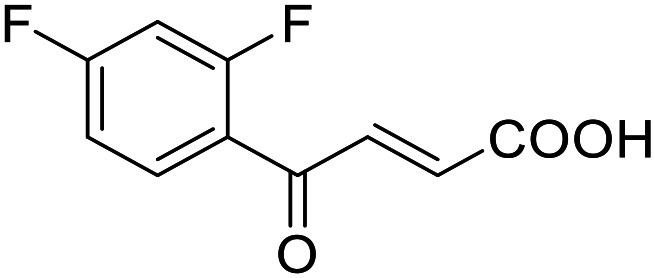	10 ± 3 μM	51 ± 9 μM	Inactive	Inactive
		(1.7 ± 0.1-fold)	(11.0 ± 0.5-fold)	(1–300 μM)	(1–300 μM)
2.43	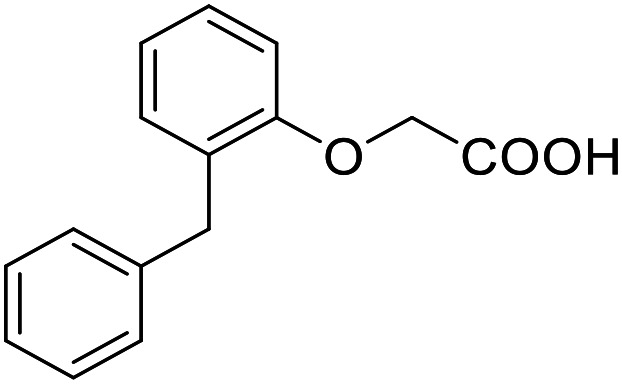	Inactive	47 ± 3 μM	Inactive	Inactive
		(1–300 μM)	(17.4 ± 0.3-fold)	(1–300 μM)	(1–300 μM)

a NR modulation was determined in uniform Gal4-hybrid reporter gene assays. Fold activation refers to the maximum reporter activation compared to DMSO (0.1%) treated cells. Data are the mean ± S.E.M.; *n* ≥ 3.

Both screening stages resulted in attractive fragment-like multi-target pharmacophores for further optimization to potent dual/multiple ligands. Among them, the phenyloxobutenoic acid scaffold (2.7, 2.16 and 2.28) showed potential on all receptors of interest with the pan-agonist 2.7 but also a tendency to selective THRα and PPARγ agonism (2.28). This dual activity profile might valuably combine therapeutic effects in liver (THR) and adipose tissue (PPARγ) to counteract steatohepatitis. Hence, we engaged on this chemotype for further SAR exploration (Tables 3 and 4).

**Table 3:** SAR evaluation of 5-phenylfuran-3-carboxylic acid scaffold on the targets of interest

ID	Structure	EC_50_^a^ (max. fold activation)			
		THRα	PPARγ	FXR	RXRα
2.7	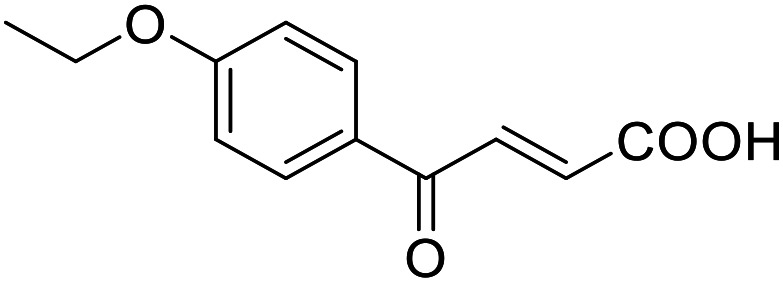	27 ± 1 μM	25.9 ± 0.4 μM	26 ± 1 μM	33 ± 6 μM
		(8.9 ± 0.5-fold)	(31.2 ± 0.6-fold)	(9.5 ± 0.4-fold)	(8 ± 1-fold)
3.1	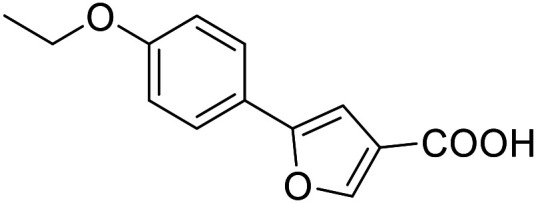	1.5 ± 0.2 μM	3 ± 1 μM	1.3 ± 0.3 μM	0.9 ± 0.2 μM
		(3.7 ± 0.2-fold)	(6.4 ± 0.6-fold)	(6.4 ± 0.3-fold)	(4.3 ± 0.5-fold)
2.16	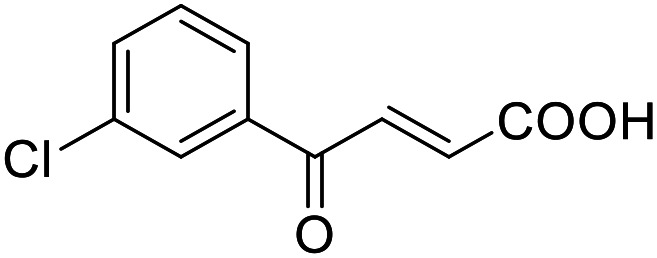	Inactive	34 ± 7 μM	Inactive	Inactive
		(1–300 μM)	(8.2 ± 0.5-fold)	(1–300 μM)	(1–300 μM)
3.2	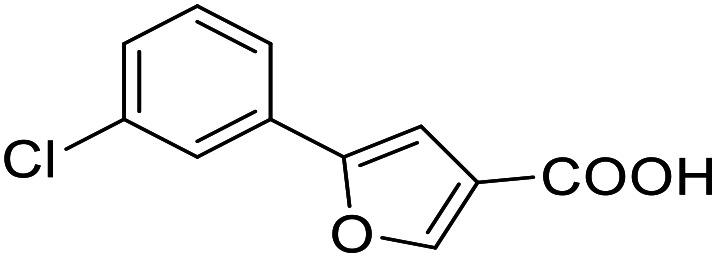	26 ± 5 μM	32 ± 2 μM	11 ± 2 μM	14 ± 5 μM
		(2.1 ± 0.2-fold)	(3.2 ± 0.2-fold)	(1.7 ± 0.1-fold)	(2.1 ± 0.2-fold)

a NR modulation was determined in uniform Gal4-hybrid reporter gene assays. Fold activation refers to the maximum reporter activation compared to DMSO (0.1%) treated cells. Data are the mean ± S.E.M.; *n* ≥ 3.

**Table 4:** SAR evaluation of 3-benzoylacrylic acid scaffold on the targets of interest

ID	Structure	EC_50_^a^ (max. fold activation)			
		THRα	PPARγ	FXR	RXRα
4.1	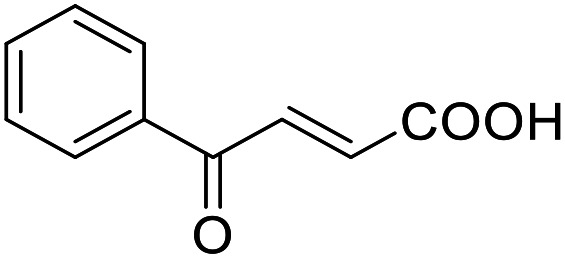	Inactive	62 ± 2 μM	Inactive	Inactive
		(1–300 μM)	(26 ± 2-fold)	(1–300 μM)	(1–300 μM)
4.2	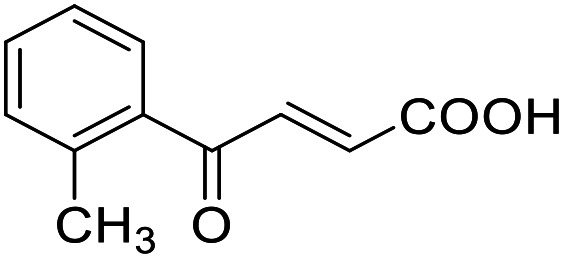	31 ± 7 μM	32 ± 1 μM	39 ± 3 μM	50 ± 4 μM
		(2.4 ± 0.3-fold)	(20 ± 1-fold)	(3.8 ± 0.4-fold)	(7 ± 2-fold)
4.3	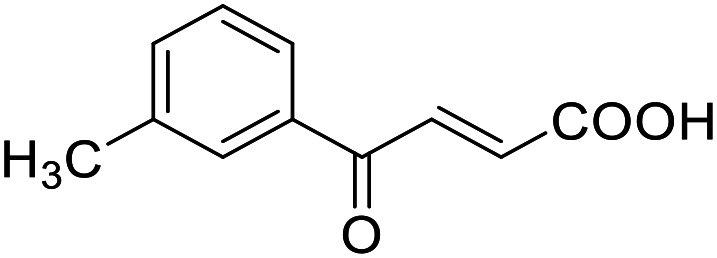	19 ± 8 μM	38 ± 1 μM	22 ± 5 μM	30 ± 5 μM
		(3.1 ± 0.6-fold)	(19 ± 1-fold)	(3.4 ± 0.4-fold)	(4.7 ± 0.5-fold)
4.4	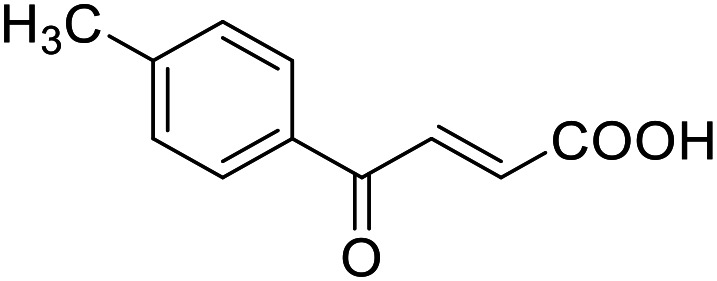	28 ± 3 μM	37 ± 1 μM	27 ± 9 μM	35 ± 1 μM
		(3.4 ± 0.4-fold)	(17.5 ± 0.9-fold)	(3.4 ± 0.7-fold)	(5.3 ± 0.2-fold)
2.16	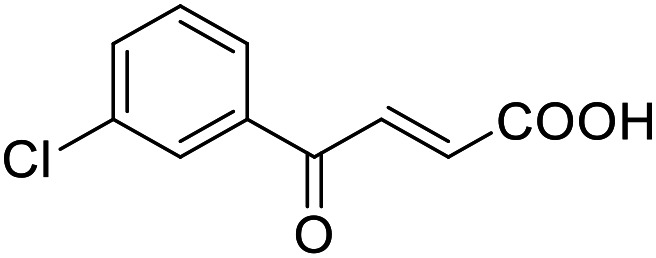	Inactive	34 ± 7 μM	Inactive	Inactive
		(1–300 μM)	(8.2 ± 0.5-fold)	(1–300 μM)	(1–300 μM)
4.5	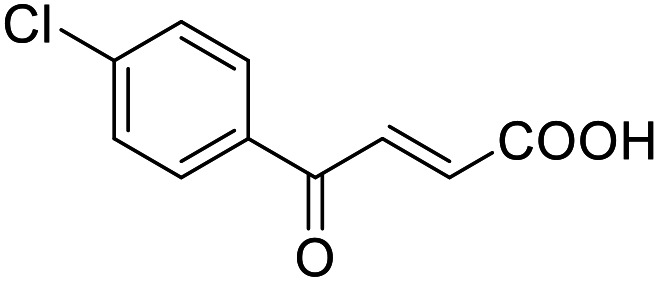	46 ± 7 μM	30 ± 4 μM	40 ± 3 μM	46 ± 1 μM
		(7 ± 2-fold)	(12 ± 2-fold)	(3.1 ± 0.3-fold)	(4.0 ± 0.2-fold)
4.6	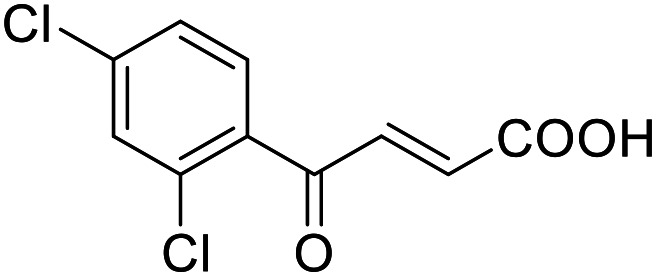	43 ± 3 μM	20 ± 5 μM	Inactive	Inactive
		(4.2 ± 0.3-fold)	(13 ± 2-fold)	(1–300 μM)	(1–300 μM)
4.7	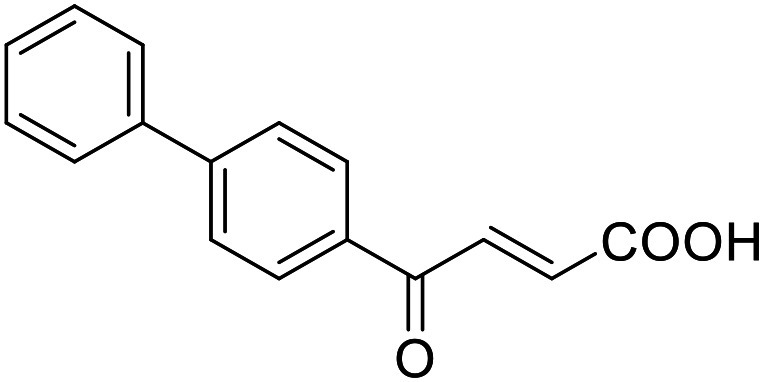	16 ± 7 μM	18 ± 4 μM	Inactive	Inactive
		(14.8 ± 1.0-fold)	(33.4 ± 2.7-fold)	(1–300 μM)	(1–300 μM)
4.8	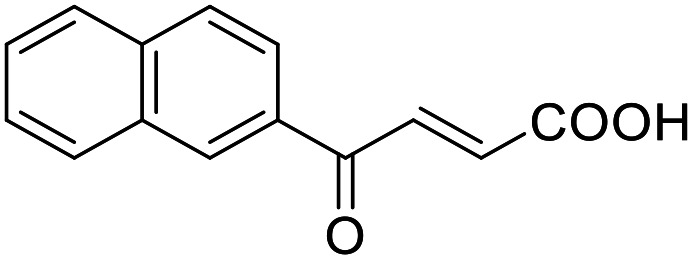	39 ± 1 μM	27 ± 1 μM	26 ± 4 μM	26 ± 3 μM
		(28 ± 1-fold)	(16.7 ± 0.5-fold)	(7 ± 1-fold)	(4.5 ± 0.5-fold)

a NR modulation was determined in uniform Gal4-hybrid reporter gene assays. Fold activation refers to the maximum reporter activation compared to DMSO (0.1%) treated cells. Data are the mean ± S.E.M.; *n* ≥ 3.

The saturated phenyloxobutanoic acid analogues of 2.7, 2.16 and 2.28 were inactive on all receptors of interest (not shown) confirming the preference for the phenyloxobutenoic acid motif. Although the γ-carbonyl group stabilizes the acrylic acid and diminishes its reactive character, we next evaluated the possibility to mimic and further stabilize the motif by incorporation in a furan ring (Table 3). The furan analogue 3.1 of the fragment hit 2.7 indeed retained agonist activity on all receptors of interest and even gained in potency. Although activation efficacy of 3.1 and the furan counterpart 3.2 of screening hit 2.16 was diminished the furan-3-carboxylate may valuably replace the oxobutenoic acid substructure in optimized derivatives.

Building on the promising dual PPARγ/THRα agonist activity of 2.7 and 2.28, we aimed to obtain an improved dual agonist with enhanced efficacy and performed a preliminary SAR study on this chemotype (Table 4). Phenyloxobutenoic acid (4.1) lacking substituents on the phenyl ring only retained PPARγ agonism while methylation in 2-, 3-, or 4-position (4.2–4.4) reinstalled activity on all receptors of interest with the weakest potency for the 2-methyl analogue (4.2). Further comparison of the 3- (2.16) and 4-chloro analogues (4.5) indicated that albeit PPARγ tolerated substituents in all positions, THRα agonism favored 4-substitution. Moreover, the preliminary SAR insights indicated that a 2-substituent might promote selectivity (2.43, 4.2). Hence, we tested the combination of 4- and 2-chloro substituents (4.6) which indeed selectively activated PPARγ and THRα with moderate potency. Introduction of a bulky phenyl substituent in 4-position (4.7) was more productive and boosted PPARγ and THRα agonism, while the similarly bulky and lipophilic β-naphthyl analogue 4.8 activated all studied receptors with considerable efficacy. Despite moderate potency, 4.6 and 4.7 hence emerged as PPARγ/THRα ligand pharmacophore for the development of potent dual agonists.

**Fig. 7:**
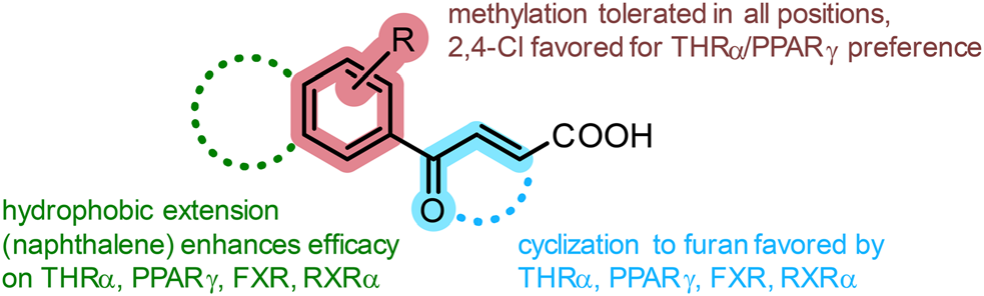
Summarized SAR of multi-target fragments based on the privileged phenyloxobutenoic acid motif.

Focused fragment screening followed by preliminary structural optimization yielded four lead pharmacophores (3.1, 4.6, 4.7, 4.8) for designed polypharmacology. In line with the hydrophobic nature of the targets' binding sites (Fig. 1), the privileged phenyloxobutenoic acid hit could be extended with lipophilic motifs and substituents for enhanced potency and efficacy. Additionally, cyclization of the oxobutenoic acid motif to a furan-3-carboxylate enhanced potency on the targets of interest but diminished efficacy. Using these preliminary SAR insights (Fig. 7) and the most active fragments as starting points for further focused screening and/or systematic structural extension may be a fruitful avenue towards potent dual ligands of the targets of interest.

**Fig. 8:**
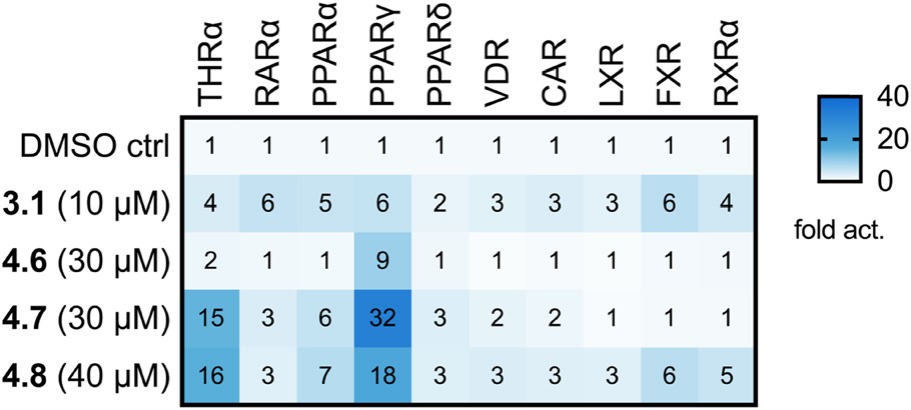
Selectivity testing of multi-target nuclear receptor modulators 3.1, 4.6, 4.7, and 4.8 on related targets. The heatmap shows the mean fold activation of the respective nuclear receptors in uniform Gal4-hybrid reporter gene assays; *n* ≥ 3. Reference ligands are described in the experimental section.

The multi-target fragment ligands 3.1, 4.6, 4.7, and 4.8 comprise low molecular weight (MW 226–252) and lipophilicity (AlogP 2.95–3.41)^39^ and can thus be substantially modified and extended within the rule-of-five^40^ during further optimization as dual/multiple ligands. With respect to its fragment character, 3.1 emerged as a considerably potent pan-agonist on THRα, PPARγ, FXR and RXRα and may serve as a valuable lead for dual ligand design for any combination of these receptors. Similarly, 4.8 activated all targets of interest and displayed substantially higher efficacy than 3.1 but also increased lipophilicity. 4.6 and 4.7, in contrast, already exhibit dual agonism on PPARγ/THRα and can be considered as starting points to optimize this activity profile. Selectivity testing of these multiple nuclear receptor ligand fragments at concentrations at or above their EC_50_ values for the targets of interest (Fig. 8) revealed further activities on related receptors. 3.1 also activated the retinoic acid receptor (RAR) α and PPARα, and to a lesser extent the vitamin D receptor (VDR) and the constitutive androstane receptor (CAR). 4.6, 4.7 and 4.8 displayed higher selectivity, but the scaffold also exhibited PPARα agonism and slight RARα activation. Given the inverse correlation between molecular size and promiscuity^37^ and the structural similarity of lipid-sensing nuclear receptor binding sites, the incomplete selectivity of the fragment hits is expectable, and extensive optimization will be needed to design dual ligands hitting only selected targets of interest. The preference observed, *e.g.*, for 4.7 and 4.8 nevertheless indicates that selective dual targeting can be achieved with these fragments as starting points.

## Conclusion

Designed polypharmacology may benefit the treatment of multifactorial diseases by exploiting synergies of simultaneous modulation of more than one dysregulated pathway.^5,41,42^ The development of dual/multiple ligands is challenging, however, as such compounds must fulfill the structural requirements for ligand binding of each target of interest.^1,3^ Fragments typically present lower selectivity^43^ and fragment screening may therefore offer access to starting points for designed multitarget ligands. Based on the ability of many nuclear receptors involved in metabolic regulation to bind fatty acid metabolites and other lipids, we here followed a focused fragment screening approach and discovered several small fatty acid mimetic scaffolds as dual/multiple nuclear receptor modulators. These hits can now enter systematic optimization approaches, and the broad and comprehensive screening dataset is an asset for data-driven drug design such as generative deep learning.

### Chemistry

**Scheme 1:**
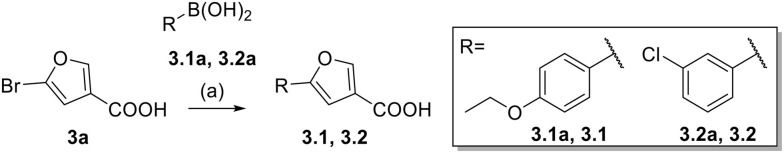
Synthesis of 3.1 and 3.2. Reagents & conditions: (a) K_2_CO_3_, XPhos Pd G2, 1,4-dioxane : H_2_O, reflux, 20 h, 80–88%.

**Scheme 2:**
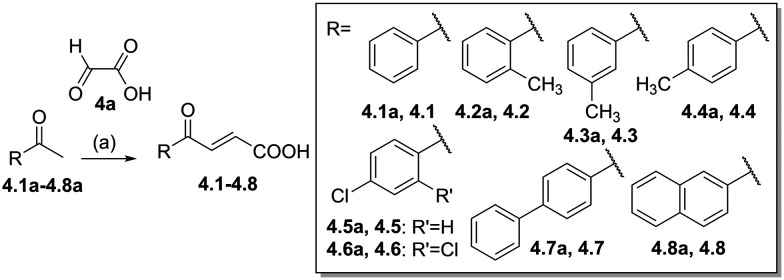
Synthesis of 4.1–4.8. Reagents & conditions: (a) AcOH : HCl, reflux, 18 h, 48–90%.

Compounds 3.1 and 3.2 were prepared *via* Suzuki coupling of 5-bromofuran-3-carboxylic acid (3a) and the corresponding boronic acids 3.1a and 3.2a (Scheme 1). Compounds 4.1–4.8 were prepared according to Scheme 2 by reacting the corresponding benzaldehydes 4.1a–4.8a with glyoxylic acid (4a) in an aldol condensation reaction.

## Experimental procedures

### Chemistry

#### General

All chemicals were of reagent grade, purchased from commercial sources (*e.g.*, Sigma-Aldrich, TCI, BLDpharm) and used without further purification unless otherwise specified. All reactions were conducted under nitrogen or argon atmosphere and in absolute solvents purchased from Sigma-Aldrich. Other solvents, especially for work-up procedures, were of reagent grade or purified by distillation (iso-hexane, cyclohexane, ethyl acetate, EtOH). Reactions were monitored by thin layer chromatography (TLC) on TLC Silica gel 60 F254 coated aluminum sheets by Merck and visualized under ultraviolet light (254 nm) or by using ninhydrin or Ehrlichs reagent stains. Purification by column chromatography was performed on a puriFlash® XS520Plus system (Advion, Ithaca, NY, USA) using high performance spherical silica columns (SIHP, 50 μM) by Interchim and a gradient of iso-hexane or cyclohexane to ethyl acetate, reversed-phase column chromatography was performed on a puriFlash® 5.250 system (Advion) using C18HP columns (SIHP, 15 μM) by Interchim and a gradient of H_2_O with 10% MeCN to 100% MeCN (HPLC gradient grade). Mass spectra were obtained on a puriFlash®-CMS system (Advion) using atmospheric pressure chemical ionization (APCI). HRMS were obtained with a Thermo Finnigan LTQ FT instrument for electron impact ionization (EI) or electrospray ionization (ESI). NMR spectra were recorded on Bruker Avance III HD 400 MHz or 500 MHz spectrometers equipped with a CryoProbe™ Prodigy broadband probe (Bruker). Chemical shifts are reported in *δ* values (ppm), coupling constants (*J*) in hertz (Hz). The purity of the compounds was determined by ^1^H NMR (qHNMR) according to the method described by Pauli *et al.*^44^ with internal calibration. To ensure accurate determination of peak area ratio, the qHNMR measurements were conducted under conditions allowing for complete relaxation. Ethyl 4-(dimethylamino)benzoate (LOT#BCCC6657, purity 99.63%) and dimethyl terephthalate (LOT#BCBT9974, purity 99.95%) were used as internal standards in CDCl_3_ or DMSO-d_6_. All compounds for biological testing had a purity >95% according to quantitative ^1^H NMR (qHNMR).

#### General procedures

##### General procedure 1 (**GP1**)

Under inert atmosphere, 5-bromofuran-3-carboxylic acid (3a, 1.0 mmol, 1.0 eq.) potassium carbonate (2.5 mmol, 2.5 eq.) and the respective boronic acid (3.1a, 3.2a, 1.2 mmol, 1.2 eq.) were suspended in a mixture of 1,4-dioxane and H_2_O (85 : 15, 0.05 M). The mixture was degassed by purging with nitrogen for 10 min. XPhos Pd G2 (0.05 mmol, 0.05 eq.) was then added, and the mixture was stirred at 95 °C for 20 h. After the reaction was completed, as monitored by TLC, the mixture was cooled to rt, acidified with 2 M HCl solution and extracted with EtOAc (3 × 20 mL). The combined organic layers were dried over MgSO_4_. The solvent was removed *in vacuo* and the residue was purified by automated flash column chromatography (cyclohexane/EtOAc 94 : 6 over 12 CV) to obtain title compound 3.1 and 3.2.

##### General procedure 2 (**GP2**)

Glyoxylic acid monohydrate (4a, 4.5 mmol, 1.5 eq.) and the respective acetophenone (4.1a–4.8a, 3.0 mmol, 1.0 eq.) were added in sequence to a solution of conc. HCl (0.5 mL) in AcOH (10 mL). The mixture was stirred under reflux for 4 h. After the reaction was completed, as monitored by TLC, the mixture was cooled to rt and dried under vacuum. The residue was dissolved in K_2_CO_3_ (25% aq.) and washed with DCM (5 × 20 mL). Then, the aqueous phase was cooled in an ice-bath and acidified with conc. HCl. The participated oil was extracted with EtOAc, the organic phase was dried over MgSO_4_, and the solvent was removed under reduced pressure. The residue was purified by reverse phase column chromatography (H_2_O/MeCN 95 : 5 → 0 : 100 over 12 CV) to obtain the title compound 4.1–4.8.

###### 5-(4-Ethoxyphenyl)furan-3-carboxylic acid (3.1)

Preparation according to GP1 using 5-bromofuran-3-carboxylic acid (3a, 346 mg, 1.0 mmol, 1.0 eq.), potassium carbonate (346 mg, 2.5 mmol, 2.5 eq.) and 4-ethoxyphenylboronic acid (3.1a, 199 mg, 1.2 mmol, 1.2 eq.) in 1,4-dioxane and H_2_O (85 : 15, 20 mL). XPhos Pd G2 (39.3 mg, 0.05 mmol, 0.05 eq.) was added. Reverse phase column chromatography (H_2_O/MeCN 95 : 5 → 0 : 100 over 12 CV) yielded the title compound 3.1 (186 mg, 0.801 mmol, yield: 80%) as a pale-yellow solid. ^1^H NMR (500 MHz, DMSO-*d*_6_) *δ* = 12.70 (s, 1H), 8.28 (d, *J* = 0.8 Hz, 1H), 7.68–7.65 (m, 2H), 7.04 (d, *J* = 0.8 Hz, 1H), 7.00–6.97 (m, 2H), 4.06 (q, *J* = 7.0 Hz, 2H), 1.33 (t, *J* = 6.9 Hz, 3H) ppm. ^13^C NMR (126 MHz, DMSO-*d*_6_) *δ* = 163.9, 158.6, 154.5, 146.9, 125.4, 122.2, 121.6, 114.8, 103.4, 63.2, 14.6 ppm. HRMS (ESI−): *m*/*z* calculated 231.0663 for [C_13_H_11_O_4_]^−^; found: 231.0663 ([M]^−^).

###### 5-(3-Chlorophenyl)furan-3-carboxylic acid (3.2)

Preparation according to GP1 using 5-bromofuran-3-carboxylic acid (3a, 346 mg, 1.0 mmol, 1.0 eq.), potassium carbonate (346 mg, 2.5 mmol, 2.5 eq.) and 3-chlorophenylboronic acid (3.2a, 188 mg, 1.2 mmol, 1.2 eq.) in 1,4-dioxane and H_2_O (85 : 15, 20 mL). XPhos Pd G2 (39.3 mg, 0.05 mmol, 0.05 eq.) was added. Reverse phase column chromatography (H_2_O/MeCN 95 : 5 → 0 : 100 over 12 CV) yielded the title compound 3.2 (197 mg, 0.887 mmol, yield: 88%) as a yellow solid. ^1^H NMR (500 MHz, DMSO-*d*_6_) *δ* = 8.39 (d, *J* = 0.8 Hz, 1H), 7.83 (t, *J* = 1.9 Hz, 1H), 7.73 (dt, *J* = 7.7, 1.3 Hz, 1H), 7.48 (t, *J* = 7.9 Hz, 1H), 7.40 (ddd, *J* = 8.1, 2.1, 1.0 Hz, 1H), 7.37 (d, *J* = 0.8 Hz, 1H) ppm. ^13^C NMR (126 MHz, DMSO-*d*_6_) *δ* = 163.6, 152.7, 148.3, 133.9, 131.4, 130.9, 127.9, 123.4, 122.3, 121.8, 106.9 ppm. HRMS (ESI−): *m*/*z* calculated 177.0107 for [C_10_H_6_OCl]^−^; found: 177.0114 ([M–COOH]^−^).

###### (*E*)-4-Oxo-4-phenylbut-2-enoic acid (4.1)

Preparation according to GP2 using glyoxylic acid monohydrate (4a, 666 mg, 4.5 mmol, 1.5 eq.) and acetophenone (4.1a, 360 mg, 3.0 mmol, 1.0 eq.) in conc. HCl (0.5 mL) in AcOH (10 mL). The mixture was stirred under reflux for 4 h. Reverse phase column chromatography (H_2_O/MeCN 95 : 5 → 0 : 100 over 12 CV) yielded the title compound 4.1 (380 mg, 2.16 mmol, yield: 72%) as a light-yellow solid. ^1^H NMR (500 MHz, DMSO-*d*_6_) *δ* = 13.15 (s, 1H), 8.05–8.00 (m, 2H), 7.87 (d, *J* = 15.6 Hz, 1H), 7.73–7.68 (m, 1H), 7.60–7.55 (m, 2H), 6.67 (d, *J* = 15.6 Hz, 1H) ppm. ^13^C NMR (126 MHz, DMSO-*d*_6_) *δ* = 189.6, 166.3, 136.2, 136.2, 134.0, 132.9, 129.1, 128.8 ppm. HRMS (ESI−): *m*/*z* calculated 175.0401 for [C_10_H_7_O_3_]^−^; found: 175.0402 ([M–H]^−^).

###### (*E*)-4-Oxo-4-(*o*-tolyl)but-2-enoic acid (4.2)

Preparation according to GP2 using glyoxylic acid monohydrate (4a, 666 mg, 4.5 mmol, 1.5 eq.) and *o*-methylacetophenone (42a, 403 mg, 3.0 mmol, 1.0 eq.) in conc. HCl (0.5 mL) in AcOH (10 mL). The mixture was stirred under reflux for 4 h. Reverse phase column chromatography (H_2_O/MeCN 95 : 5 → 0 : 100 over 12 CV) yielded the title compound 4.2 (449 mg, 2.36 mmol, yield: 79%) as a yellow solid. ^1^H NMR (400 MHz, CDCl_3_) *δ* = 7.69 (d, *J* = 15.7 Hz, 1H), 7.60 (dd, *J* = 8.0, 1.5 Hz, 1H), 7.44 (td, *J* = 7.7, 1.4 Hz, 1H), 7.33–7.28 (m, 2H), 6.70 (d, *J* = 15.8 Hz, 1H), 2.50 (s, 3H) ppm. ^13^C NMR (101 MHz, CDCl_3_) *δ* = 193.5, 170.7, 141.8, 138.9, 136.7, 132.3, 132.2, 131.6, 129.5, 125.9, 21.1 ppm. HRMS (ESI−): *m*/*z* calculated 189.0557 for [C_11_H_9_O_3_]^−^; found: 189.0558 ([M–H]^−^).

###### (*E*)-4-Oxo-4-(*m*-tolyl)but-2-enoic acid (4.3)

Preparation according to GP2 using glyoxylic acid monohydrate (4a, 666 mg, 4.5 mmol, 1.5 eq.) and *m*-methylacetophenone (43a, 403 mg, 3.0 mmol, 1.0 eq.) in conc. HCl (0.5 mL) in AcOH (10 mL). The mixture was stirred under reflux for 4 h. Reverse phase column chromatography (H_2_O/MeCN 95 : 5 → 0 : 100 over 12 CV) yielded the title compound 4.3 (512 mg, 2.69 mmol, yield: 90%) as a pale-yellow solid. ^1^H NMR (400 MHz, CDCl_3_) *δ* = 7.99 (d, *J* = 15.5 Hz, 1H), 7.83–7.78 (m, 2H), 7.47–7.39 (m, 2H), 6.89 (d, *J* = 15.6 Hz, 1H), 2.45 (s, 3H) ppm. ^13^C NMR (101 MHz, CDCl_3_) *δ* = 189.4, 170.5, 138.9, 138.7, 136.4, 134.9, 131.2, 129.4, 128.8, 126.2, 21.4 ppm. HRMS (ESI−): *m*/*z* calculated 189.0557 for [C_11_H_9_O_3_]^−^; found: 189.0557 ([M–H]^−^).

###### (*E*)-4-Oxo-4-(*p*-tolyl)but-2-enoic acid (4.4)

Preparation according to GP2 using glyoxylic acid monohydrate (4a, 666 mg, 4.5 mmol, 1.5 eq.) and *p*-methylacetophenone (4.4a, 403 mg, 3.0 mmol, 1.0 eq.) in conc. HCl (0.5 mL) in AcOH (10 mL). The mixture was stirred under reflux for 4 h. Reverse phase column chromatography (H_2_O/MeCN 95 : 5 → 0 : 100 over 12 CV) yielded the title compound 4.4 (441 mg, 2.32 mmol, yield: 77%) as a light-yellow solid. ^1^H NMR (500 MHz, DMSO-*d*_6_) *δ* = 7.96–7.92 (m, 2H), 7.87 (d, *J* = 15.5 Hz, 1H), 7.39 (d, *J* = 7.9 Hz, 2H), 6.66 (d, *J* = 15.6 Hz, 1H), 2.40 (s, 3H) ppm. ^13^C NMR (126 MHz, DMSO-*d*_6_) *δ* = 189.4, 166.8, 145.2, 136.7, 134.2, 133.7, 130.1, 129.4, 21.7 ppm. HRMS (ESI−): *m*/*z* calculated 145.0653 for [C_10_H_9_O]^−^; found: 145.0660 ([M–COOH]^−^).

###### (*E*)-4-Oxo-4-(4-chlorophenyl)but-2-enoic acid (4.5)

Preparation according to GP2 using glyoxylic acid monohydrate (4a, 666 mg, 4.5 mmol, 1.5 eq.) and *p*-chloroacetophenone (4.5a, 464 mg, 3.0 mmol, 1.0 eq.) in conc. HCl (0.5 mL) in AcOH (10 mL). The mixture was stirred under reflux for 4 h. Reverse phase column chromatography (H_2_O/MeCN 95 : 5 → 0 : 100 over 12 CV) yielded the title compound 4.5 (301 mg, 1.43 mmol, yield: 48%) as a light-yellow solid. ^1^H NMR (400 MHz, DMSO-*d*_6_) *δ* = 13.21 (s, 1H), 8.07–8.03 (m, 2H), 7.86 (d, *J* = 15.6 Hz, 1H), 7.67–7.63 (m, 2H), 6.68 (d, *J* = 15.6 Hz, 1H) ppm. ^13^C NMR (101 MHz, DMSO-*d*_6_) *δ* = 188.6, 166.2, 138.9, 135.9, 134.9, 133.3, 130.8, 129.2 ppm. HRMS (ESI−): *m*/*z* calculated 242.9621 for [C_10_H_5_O_3_Cl_2_]^−^; found: 242.9619 ([M–H]^−^).

###### (*E*)-4-Oxo-4-(2,4-dichlorophenyl)but-2-enoic acid (4.6)

Preparation according to GP2 using glyoxylic acid monohydrate (4a, 666 mg, 4.5 mmol, 1.5 eq.) and *o,p*-dichloroacetophenone (4.6a, 567 mg, 3.0 mmol, 1.0 eq.) in conc. HCl (0.5 mL) in AcOH (10 mL). The mixture was stirred under reflux for 4 h. Reverse phase column chromatography (H_2_O/MeCN 95 : 5 → 0 : 100 over 12 CV) yielded the title compound 4.6 (411 mg, 1.68 mmol, yield: 56%) as a colorless solid. ^1^H NMR (400 MHz, DMSO-*d*_6_) *δ* = 7.81 (dd, *J* = 2.0, 0.3 Hz, 1H), 7.68 (dd, *J* = 8.3, 0.3 Hz, 1H), 7.60 (dd, *J* = 8.3, 2.0 Hz, 1H), 7.31 (d, *J* = 15.9 Hz, 1H), 6.49 (d, *J* = 15.9 Hz, 1H). ^13^C NMR (101 MHz, DMSO-*d*_6_) *δ* = 188.6, 166.2, 138.9, 135.9, 134.9, 133.3, 130.8, 129.2 ppm. HRMS (ESI−): *m*/*z* calculated 209.0011 for [C_10_H_6_O_3_Cl]^−^; found: 209.0010 ([M–H]^−^).

###### (*E*)-4-([1,1′-Biphenyl]-4-yl)-4-oxobut-2-enoic acid (4.7)

Preparation according to GP2 using glyoxylic acid monohydrate (4a, 666 mg, 4.5 mmol, 1.5 eq.) and *p*-phenylacetophenone (4.7a, 589 mg, 3.0 mmol, 1.0 eq.) in conc. HCl (0.5 mL) in AcOH (10 mL). The mixture was stirred under reflux for 4 h. Reverse phase column chromatography (H_2_O/MeCN 95 : 5 → 0 : 100 over 12 CV) yielded the title compound 4.7 (481 mg, 1.91 mmol, yield: 64%) as a beige solid. ^1^H NMR (400 MHz, DMSO-*d*_6_) *δ* = 8.13 (d, *J* = 8.4 Hz, 2H), 7.94–7.87 (m, 3H), 7.79–7.76 (m, 2H), 7.55–7.50 (m, 2H), 7.48–7.43 (m, 1H), 6.71 (d, *J* = 15.5 Hz, 1H) ppm. ^13^C NMR (101 MHz, DMSO-*d*_6_) *δ* = 189.0, 166.5, 145.3, 138.7, 135.9, 135.0, 129.5, 129.1, 128.6, 127.2, 127.1, 126.9 ppm. HRMS (ESI−): *m*/*z* calculated 251.0714 for [C_16_H_11_O_3_]^−^; found: 251.0714 ([M–H]^−^).

###### (*E*)-4-Oxo-4-(naphthalen-2-yl)but-2-enoic acid (4.8)

Preparation according to GP2 using glyoxylic acid monohydrate (4a, 666 mg, 4.5 mmol, 1.5 eq.) and 1-(naphthalen-2-yl)ethanone (4.8a, 511 mg, 3.0 mmol, 1.0 eq.) in conc. HCl (0.5 mL) in AcOH (10 mL). The mixture was stirred under reflux for 4 h. Reverse phase column chromatography (H_2_O/MeCN 95 : 5 → 0 : 100 over 12 CV) yielded the title compound 4.8 (531 mg, 2.35 mmol, yield: 78%) as a yellow solid. ^1^H NMR (500 MHz, DMSO-*d*_6_) *δ* = 13.22 (s, 1H), 8.84 (d, *J* = 1.7 Hz, 1H), 8.21 (dd, *J* = 8.2, 1.2 Hz, 1H), 8.10 (d, *J* = 15.5 Hz, 1H), 8.07 (d, *J* = 8.7 Hz, 1H), 8.02 (dd, *J* = 8.7, 1.7 Hz, 2H), 7.71 (ddd, *J* = 8.2, 6.8, 1.3 Hz, 1H), 7.65 (ddd, *J* = 8.1, 6.8, 1.3 Hz, 1H), 6.77 (d, *J* = 15.5 Hz, 1H) ppm. ^13^C NMR (126 MHz, DMSO-*d*_6_) *δ* = 189.3, 166.6, 136.3, 135.5, 133.7, 133.1, 132.4, 131.7, 130.1, 129.4, 128.9, 127.9, 127.3, 123.9 ppm. HRMS (ESI−): *m*/*z* calculated 225.0557 for [C_14_H_9_O_3_]^−^; found: 225.0558 ([M–H]^−^).

### *In vitro* characterization

#### Hybrid reporter gene assays

NR modulation was determined as described previously^45^ in Gal4 hybrid reporter gene assays in HEK293T cells (German Collection of Microorganisms and Cell Culture GmbH, DSMZ) using pFR-Luc (Stratagene, La Jolla, CA, USA; reporter), pRL-SV40 (Promega, Madison, WI, USA; internal control) and the hybrid receptor clones pFA-CMV-hTHRα-LBD,^46^ pFA-CMV-hPPARγ-LBD,^47^ pFA-CMV-hFXR-LBD,^48^ and pFA-CMV-hRXRα-LBD,^49^ coding for the hinge region and ligand binding domain of the canonical isoform of the respective NR. Cells were cultured in Dulbecco's modified Eagle's medium (DMEM), high glucose supplemented with 10% fetal calf serum (FCS), sodium pyruvate (1 mM), penicillin (100 U mL^−1^), and streptomycin (100 μg mL^−1^) at 37 °C and 5% CO_2_ and seeded in 96-well plates (3 × 10^4^ cells per well). After 24 h, medium was changed to Opti-MEM without supplements and cells were transiently transfected using Lipofectamine LTX reagent (Invitrogen, Carlsbad, CA, USA) according to the manufacturer's protocol. Five hours after transfection, cells were incubated with the test compounds in Opti-MEM supplemented with penicillin (100 U mL^−1^), streptomycin (100 μg mL^−1^) and 0.1% DMSO for 16 h before luciferase activity was measured using the Dual-Glo Luciferase Assay System (Promega) according to the manufacturer's protocol on a Tecan Spark luminometer (Tecan Deutschland GmbH, Crailsheim, Germany). Firefly luminescence was divided by Renilla luminescence and multiplied by 1000 resulting in relative light units (RLU) to normalize for transfection efficiency and cell growth. Fold activation was obtained by dividing the mean RLU of test compound by the mean RLU of the untreated control. All samples were tested in at least three biologically independent experiments in duplicates. For dose–response curve fitting and calculation of EC_50_ values, the equation “[agonist] *vs.* response − variable slope (four parameters)” was used in GraphPad Prism (version 7.00, GraphPad Software, La Jolla, CA, USA). For selectivity profiling, the hybrid reporter gene assay was performed with Gal4 fusion receptor plasmids for RARα, PPARα, PPARδ, VDR, CAR and LXRα. The following reference agonists were used: triiodothyronine (1 μM, THR), all-*trans* retinoic acid (1 μM, RAR), GW7647 (1 μM, PPARα), pioglitazone (1 μM, PPARγ), L165041 (1 μM, PPARδ), calcitriol (1 μM, VDR), CITCO (1 μM, CAR), T0901317 (1 μM, LXR), GW4064 (1 μM, FXR), bexarotene (1 μM, RXR).

#### Computational procedures

Molecular descriptors, fingerprints and flexible alignments were calculated in KNIME (v4.3.2) using RDKit software (v4.2.0).

## Abbreviations

CAR: Constitutive androstane receptor
FXR: Farnesoid X receptor
HBA: H-bond acceptor
HBD: H-bond donor
LXR: Liver X receptor
MASH: Metabolic dysfunction-associated steatohepatitis
MW: Molecular weight
NASH: Non-alcoholic steatohepatitis
NR: Nuclear receptor
PPAR: Peroxisome proliferator-activated receptor
RAR: Retinoic acid receptor
RXR: Retinoid X receptor
THR: Thyroid hormone receptor
VDR: Vitamin D receptor

## Conflicts of interest

There is no conflict of interest to declare.

## Supplementary Material

MD-016-D5MD00531K-s001

## Data Availability

The data supporting this article have been included as part of the ESI.†
