# Supplementary material for: Fragment-based discovery of dual ligand pharmacophores for lipid-sensing transcription factors for designed polypharmacology
Source: RSC Med Chem. 2025 Jul 23;16(10):5012–24. doi: 10.1039/d5md00531k (PMC12352620; doi:10.1039/d5md00531k)

Supplementary Information (SI) for RSC Medicinal Chemistry.
This journal is © The Royal Society of Chemistry 2025

- Supporting Information -

Fragment-based discovery of dual ligand pharmacophores for lipid-sensing transcription factors for designed polypharmacology

Tanja Stiller[1], Silke Duensing-Kropp[1], Julian A. Marschner[1], Daniel Merk[1]*

[1] Ludwig-Maximilians-Universität (LMU) München, Department of Pharmacy, 81377 Munich, Germany

* daniel.merk@cup.lmu.de

Table of Contents

NMR spectra of **3.1, 3.2, 4.1 – 4.8** S2

NMR spectra of 3.1, 3.2, 4.1 – 4.8

Compound **3.1**

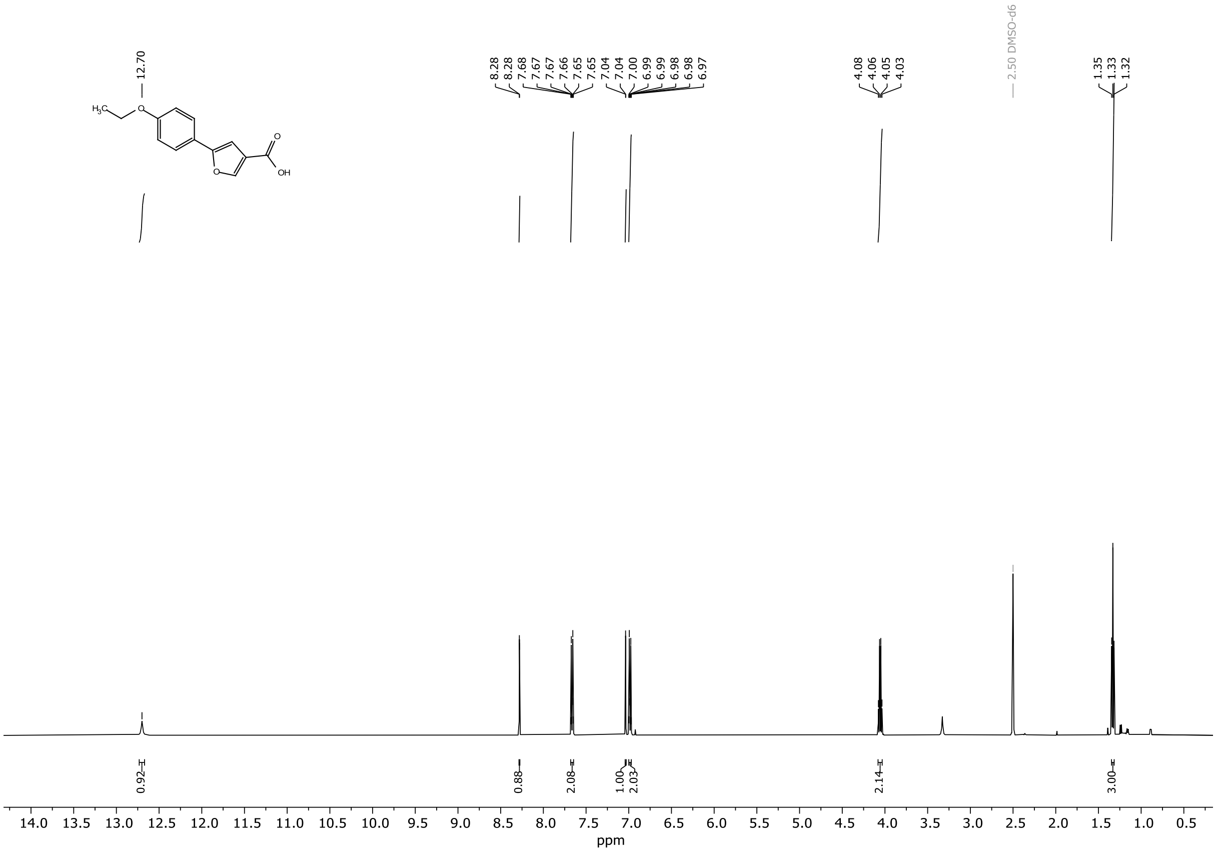

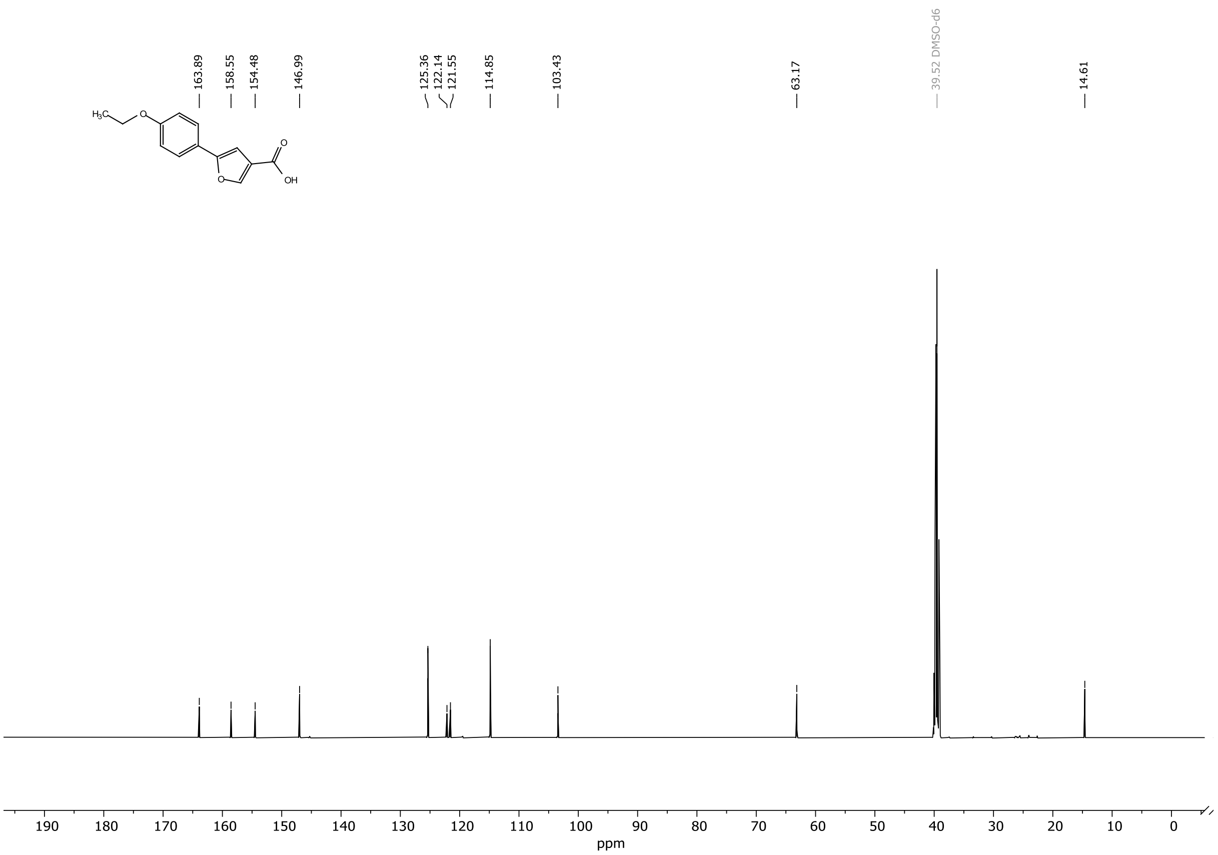

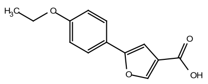

Average Purity = **95.5%**
Assuming sample weight: 3.699 mg, and mol weight: 232.24
Using Reference Compound: Ethyl 4-(dimethylamino)benzoate (2.91 mg, 99% purity, Mol Weight=193.24)
Sample Integral 1: 4.01909 - 4.09162 ppm, value = 1.02033 (2 nuclides) - Purity = 95.5%
Reference Integral: 4.18925 - 4.2471 ppm, value = 1 (2 nuclides)

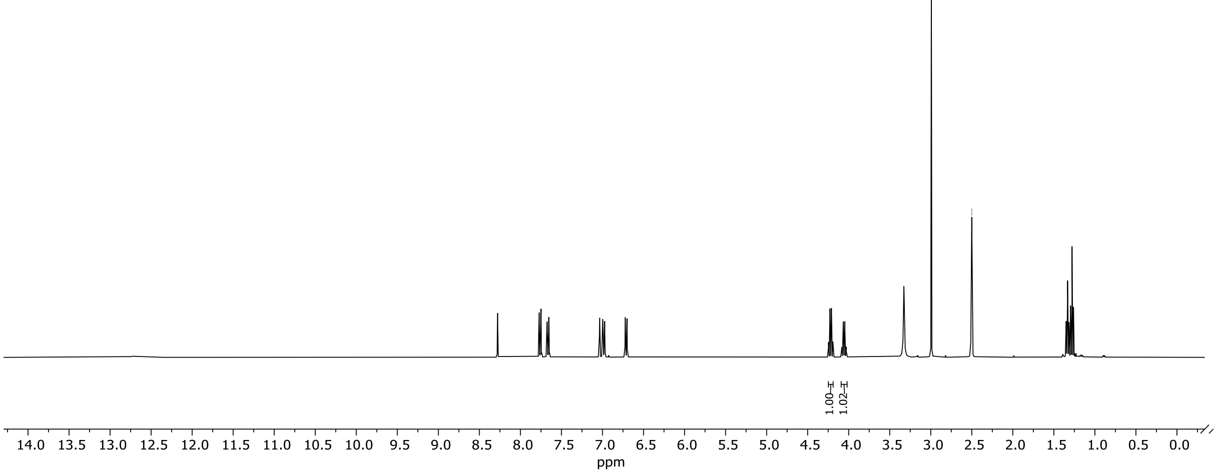

Compound **3.2**

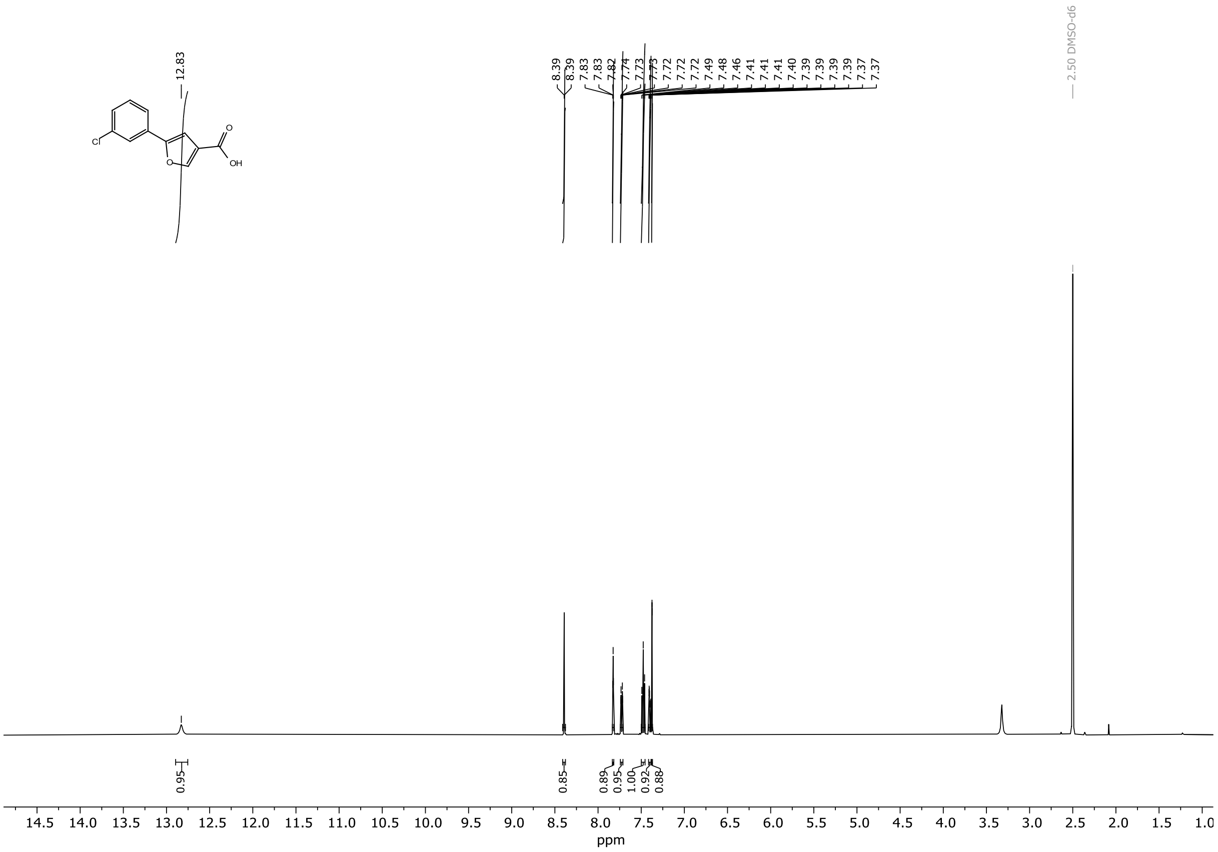

12.83
8.39
8.39
7.83
7.83
7.82
7.74
7.74
7.73
7.73
7.72
7.72
7.72
7.49
7.48
7.46
7.41
7.41
7.40
7.39
7.39
7.39
7.39
7.37
7.37
2.50 DMSO-d6
Cl
O
OH
0.95
0.85
0.89
0.95
1.00
0.92
0.88
14.5 14.0 13.5 13.0 12.5 12.0 11.5 11.0 10.5 10.0 9.5 9.0 8.5 8.0 7.5 7.0 6.5 6.0 5.5 5.0 4.5 4.0 3.5 3.0 2.5 2.0 1.5 1.0
ppm

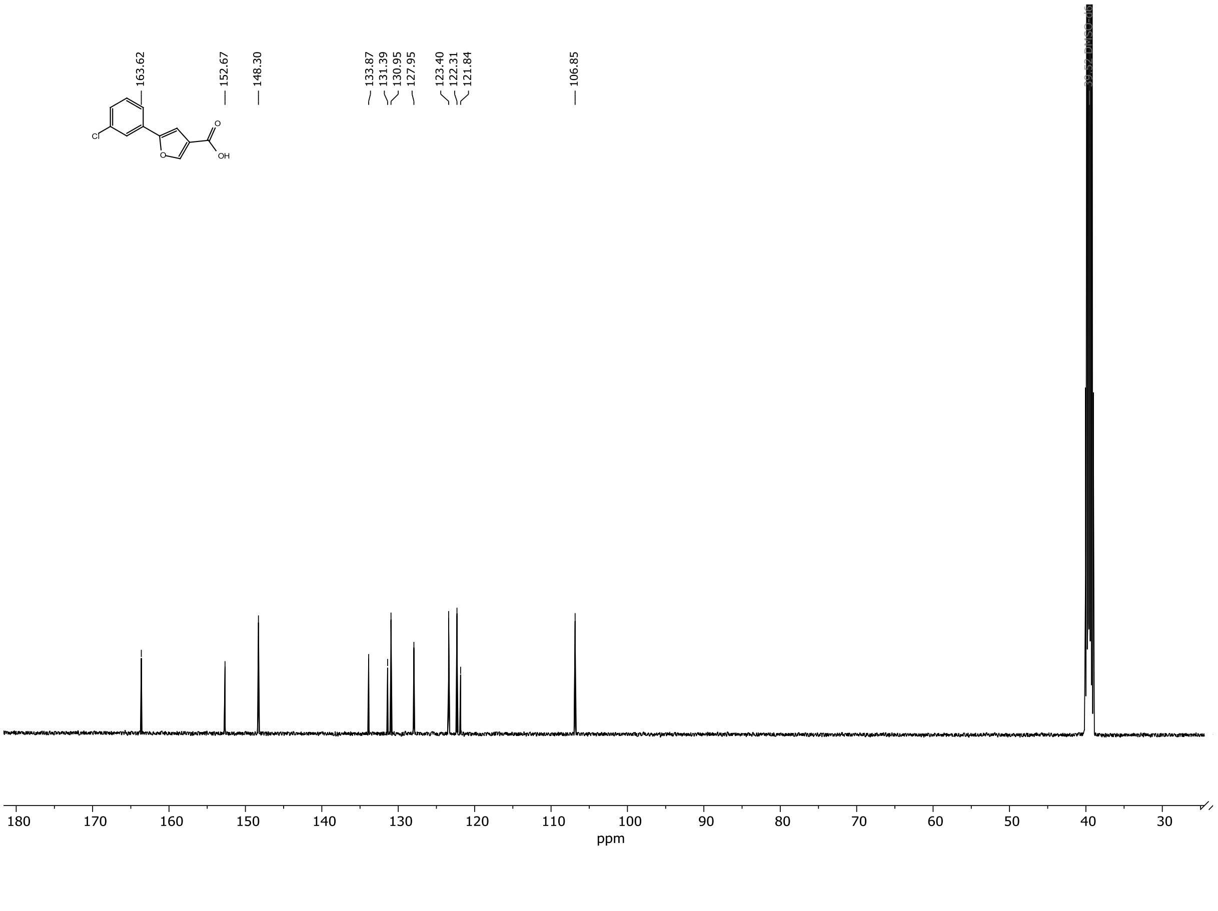

163.62
152.67
148.30
133.87
131.39
130.95
127.95
123.40
122.31
121.84
106.85
Cl
O
OH
180 170 160 150 140 130 120 110 100 90 80 70 60 50 40 30
ppm

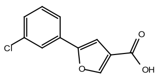

Average Purity = **97.46%**
Assuming sample weight: 1.504 mg, and mol weight: 222.6
Using Reference Compound: Ethyl 4-(dimethylamino)benzoate (1.373 mg, 99% purity, Mol Weight=193.24)
Sample Integral 1: 7.43648 - 7.51043 ppm, value = 0.46809 (1 nuclides) - Purity = 97.5%
Reference Integral: 4.19177 - 4.24736 ppm, value = 1 (2 nuclides)

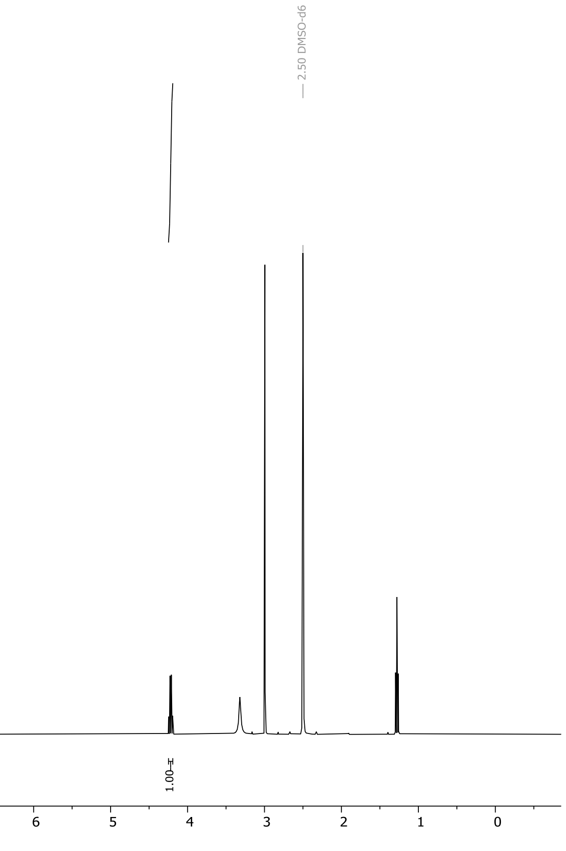

Compound **4.1**

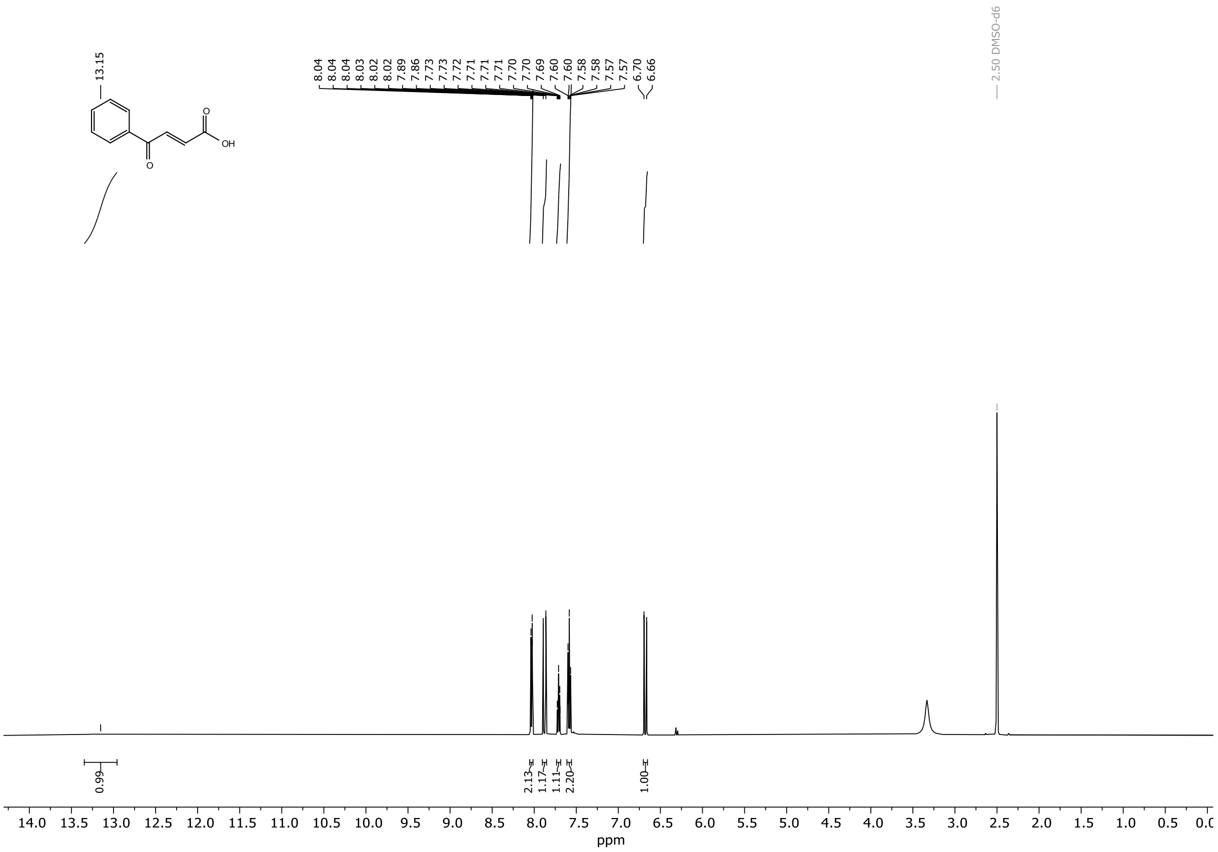
13.15
8.04
8.04
8.04
8.03
8.02
8.02
7.89
7.86
7.73
7.73
7.72
7.71
7.71
7.71
7.70
7.70
7.69
7.60
7.60
7.58
7.58
7.57
7.57
6.70
6.66
2.50 DMSO-d6
OH
O
O
0.99
2.13
1.17
1.11
2.20
1.00
14.0 13.5 13.0 12.5 12.0 11.5 11.0 10.5 10.0 9.5 9.0 8.5 8.0 7.5 7.0 6.5 6.0 5.5 5.0 4.5 4.0 3.5 3.0 2.5 2.0 1.5 1.0 0.5 0.0
ppm

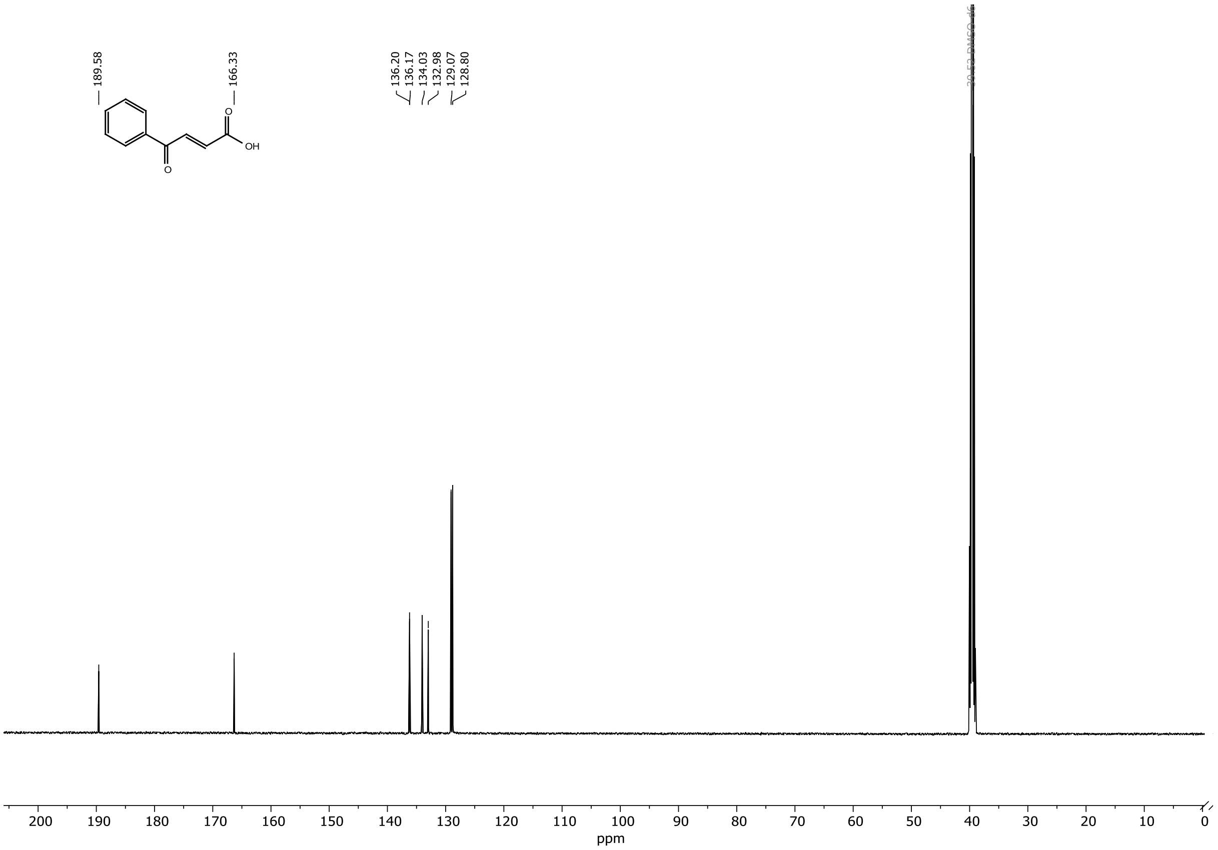
189.58
166.33
136.20
136.17
134.03
132.98
129.07
128.80
OH
O
O
200 190 180 170 160 150 140 130 120 110 100 90 80 70 60 50 40 30 20 10 0
ppm

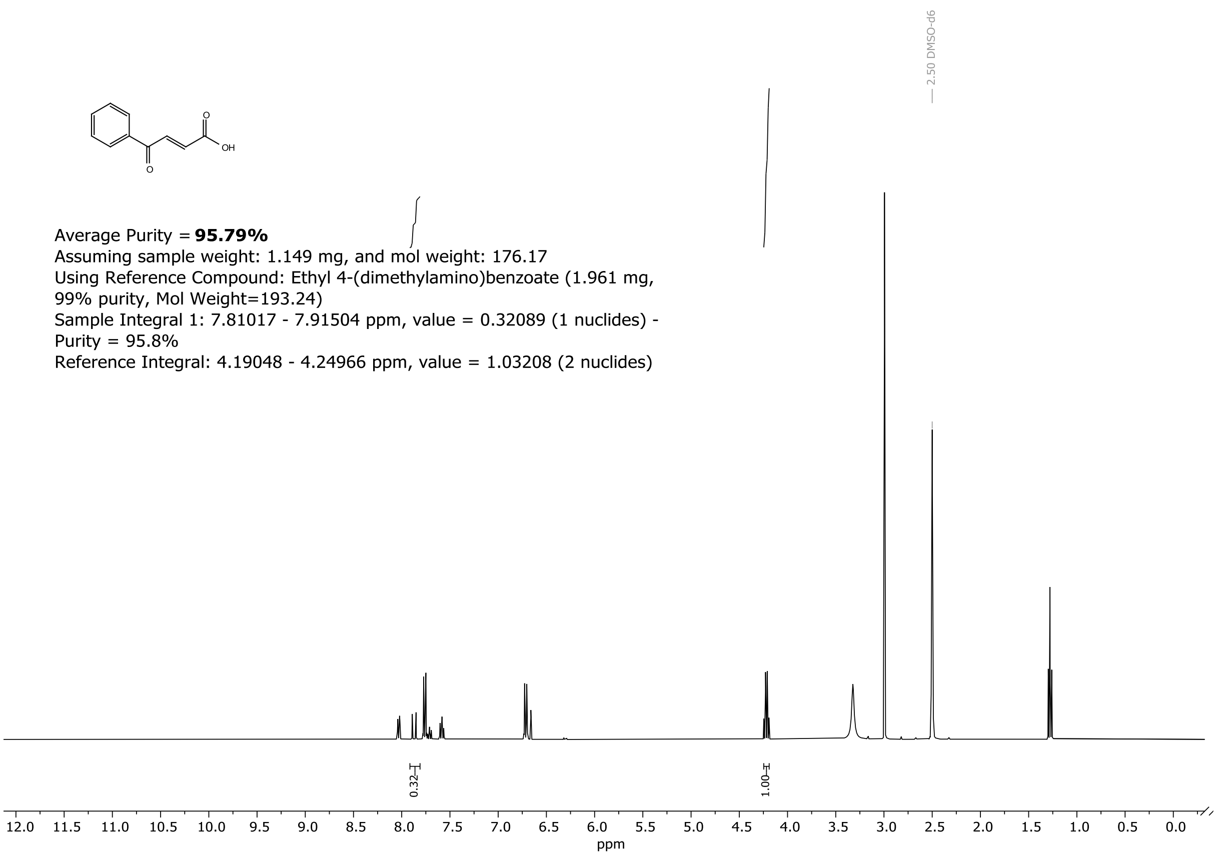

O
OH
O
2.50 DMSO-d6
Average Purity = **95.79%**
Assuming sample weight: 1.149 mg, and mol weight: 176.17
Using Reference Compound: Ethyl 4-(dimethylamino)benzoate (1.961 mg, 99% purity, Mol Weight=193.24)
Sample Integral 1: 7.81017 - 7.91504 ppm, value = 0.32089 (1 nuclides) - Purity = 95.8%
Reference Integral: 4.19048 - 4.24966 ppm, value = 1.03208 (2 nuclides)
0.32
1.00
12.0 11.5 11.0 10.5 10.0 9.5 9.0 8.5 8.0 7.5 7.0 6.5 6.0 5.5 5.0 4.5 4.0 3.5 3.0 2.5 2.0 1.5 1.0 0.5 0.0
ppm

Compound **4.2**

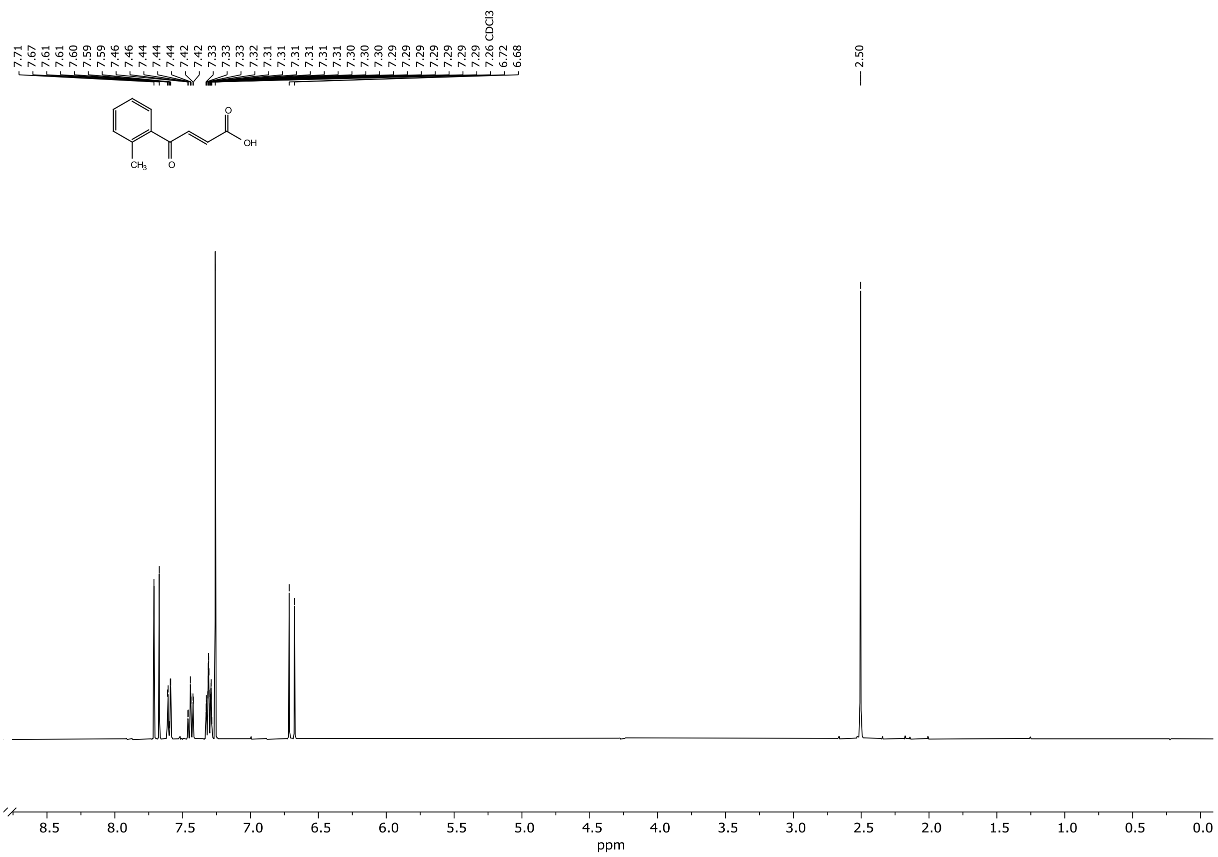
7.71
7.67
7.61
7.60
7.59
7.59
7.46
7.46
7.44
7.44
7.42
7.42
7.33
7.33
7.33
7.32
7.31
7.31
7.31
7.31
7.31
7.30
7.30
7.29
7.29
7.29
7.29
7.29
7.29
7.26 CDCl3
6.72
6.68
2.50
OH
O
CH3
ppm
8.5
8.0
7.5
7.0
6.5
6.0
5.5
5.0
4.5
4.0
3.5
3.0
2.5
2.0
1.5
1.0
0.5
0.0

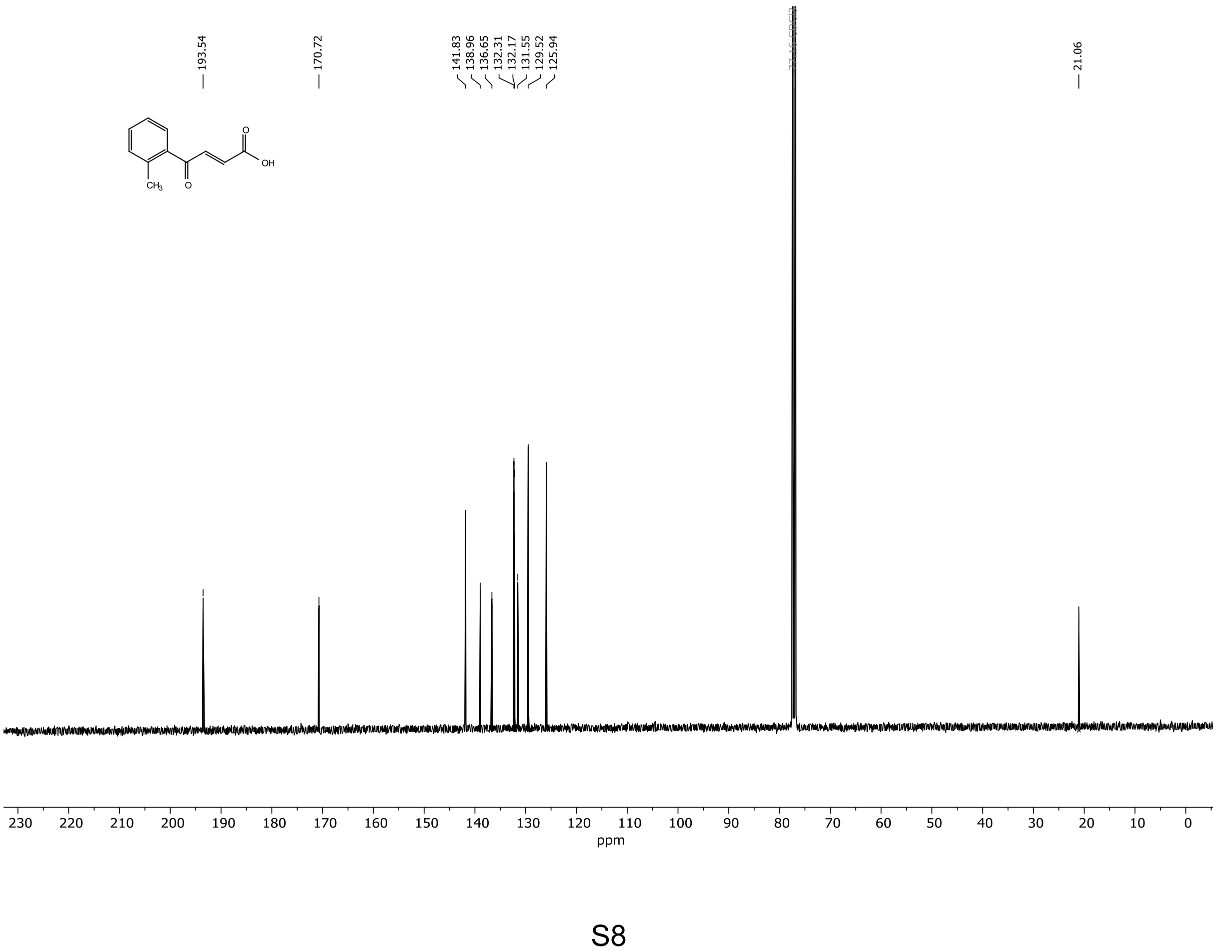
193.54
170.72
141.83
138.96
136.65
132.31
132.17
131.55
129.52
125.94
21.06
OH
O
CH3
ppm
230
220
210
200
190
180
170
160
150
140
130
120
110
100
90
80
70
60
50
40
30
20
10
0

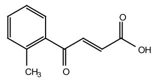

Average Purity = **95.18%**
Assuming sample weight: 1.451 mg, and mol weight: 190.2
Using Reference Compound: Dimethyl terephthalate (2.666 mg, 99% purity, Mol Weight=194.19)
Sample Integral 1: 7.40895 - 7.46485 ppm, value = 0.13356 (1 nuclides) - Purity = 95.2%
Reference Integral: 8.07735 - 8.11829 ppm, value = 1 (4 nuclides)

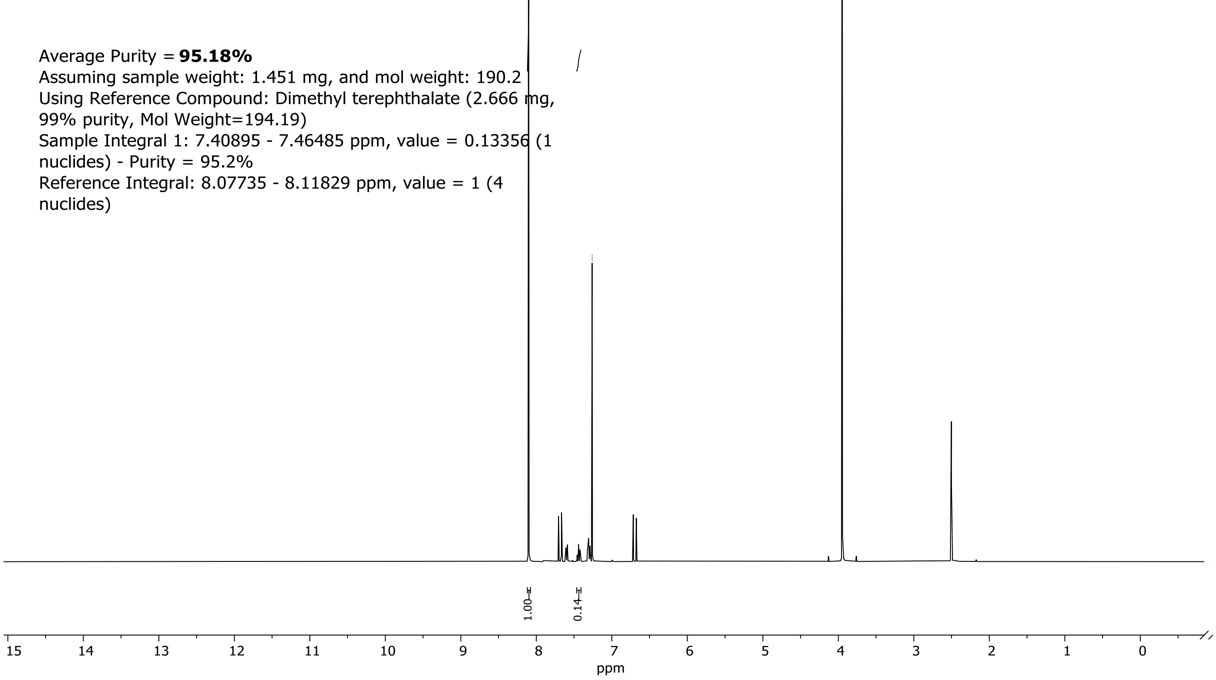

Compound **4.3**

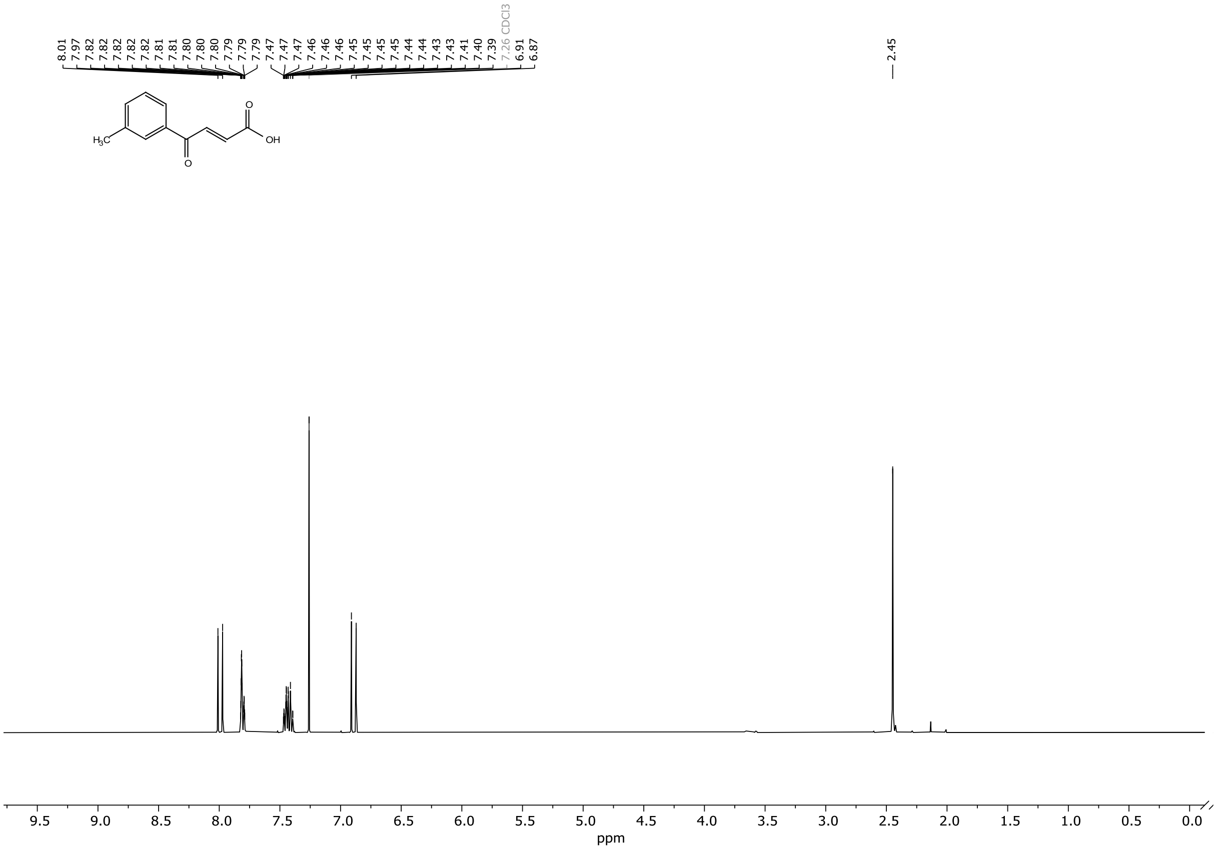
8.01
7.97
7.82
7.82
7.82
7.82
7.82
7.81
7.81
7.80
7.80
7.79
7.79
7.79
7.47
7.47
7.47
7.46
7.46
7.46
7.45
7.45
7.45
7.45
7.44
7.44
7.43
7.43
7.41
7.40
7.39
7.26 CDCl3
6.91
6.87
2.45
H3C
O
OH
ppm

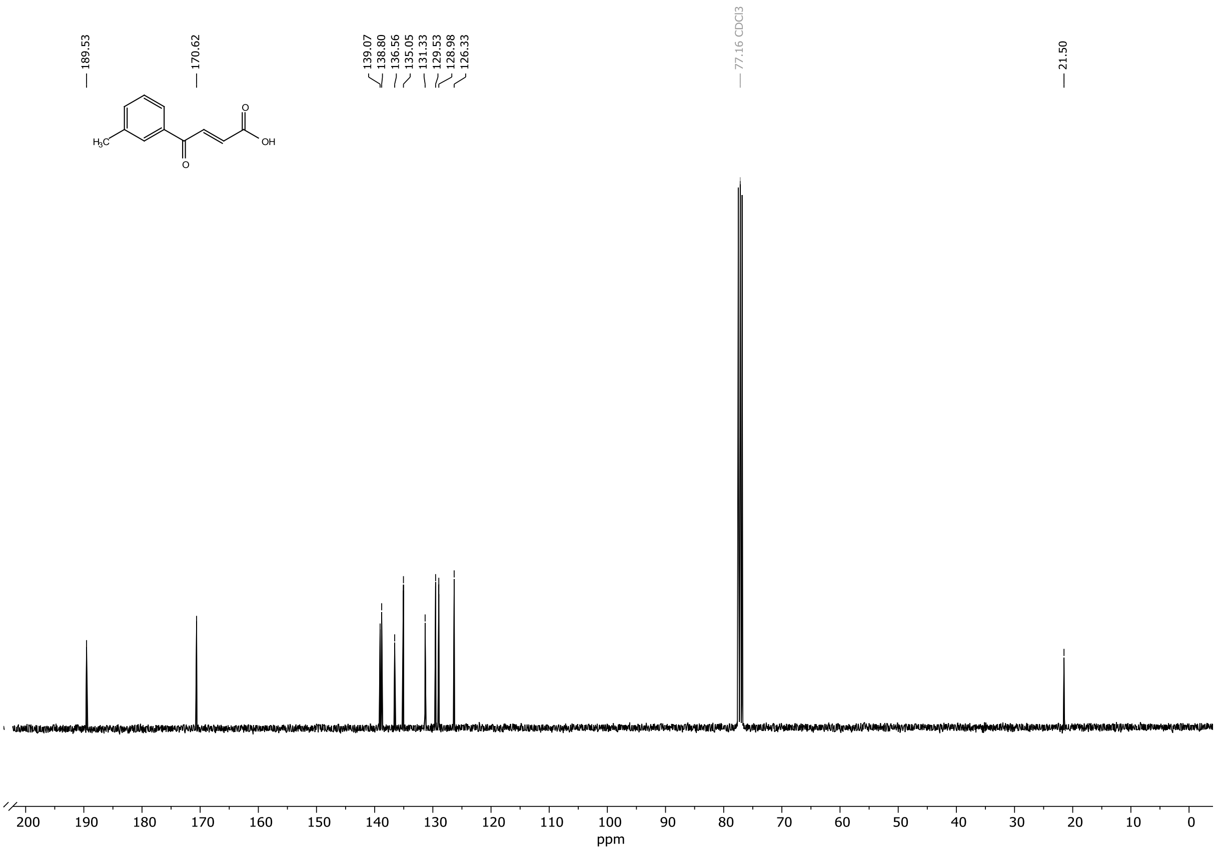
189.53
170.62
139.07
138.80
136.56
135.05
131.33
129.53
128.98
126.33
77.16 CDCl3
21.50
H3C
O
OH
ppm

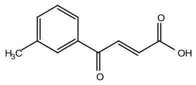

Average Purity = **96.53%**
Assuming sample weight: 3.244 mg, and mol weight: 190.2
Using Reference Compound: Ethyl 4-(dimethylamino)benzoate (2.401 mg, 99% purity, Mol Weight=193.24)
Sample Integral 1: 7.8446 - 7.91285 ppm, value = 0.65519 (1 nuclides) - Purity = 96.5%
Reference Integral: 4.1942 - 4.25518 ppm, value = 1 (2 nuclides)

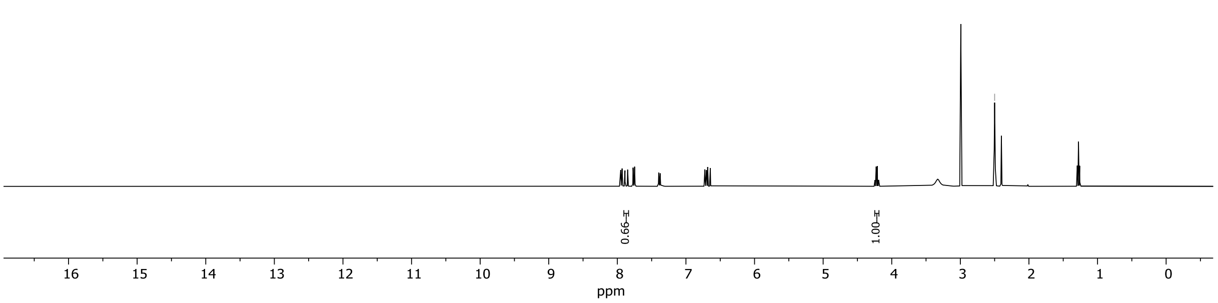

Compound **4.4**

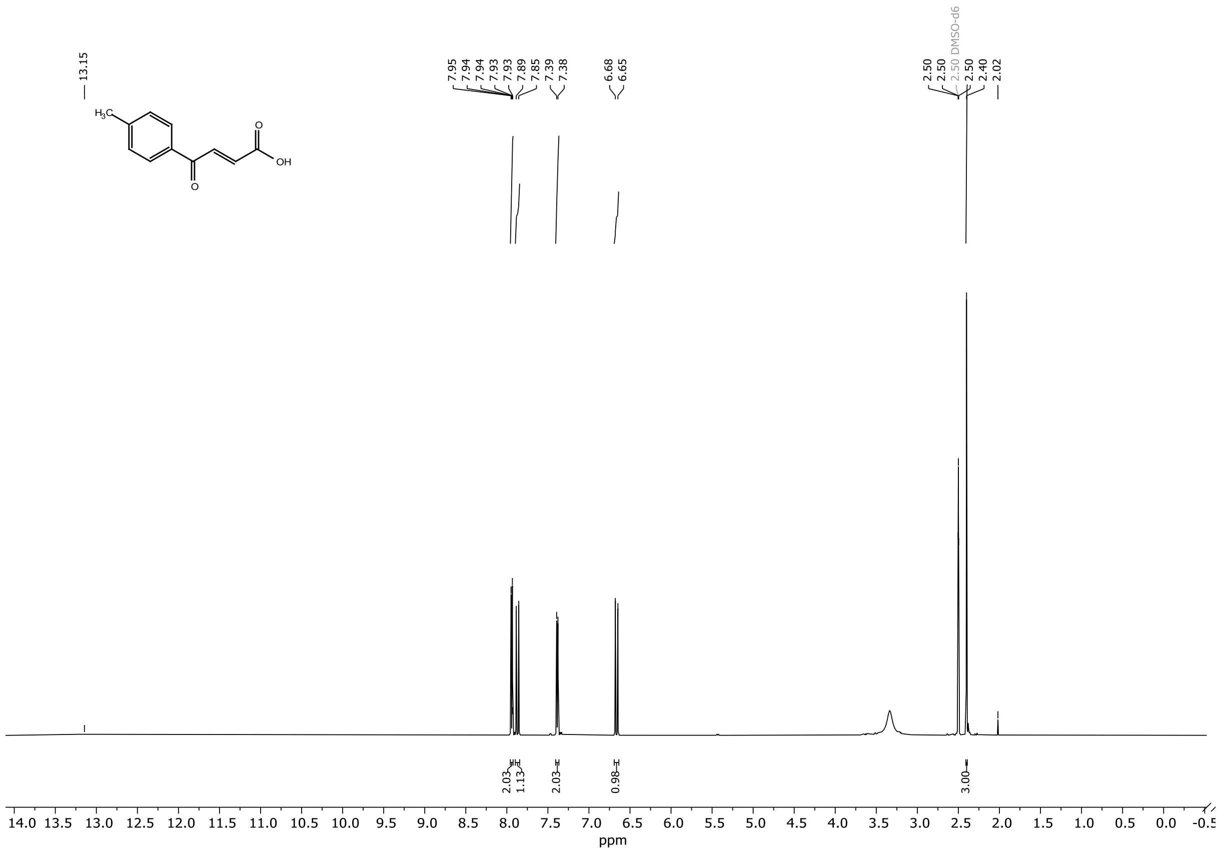

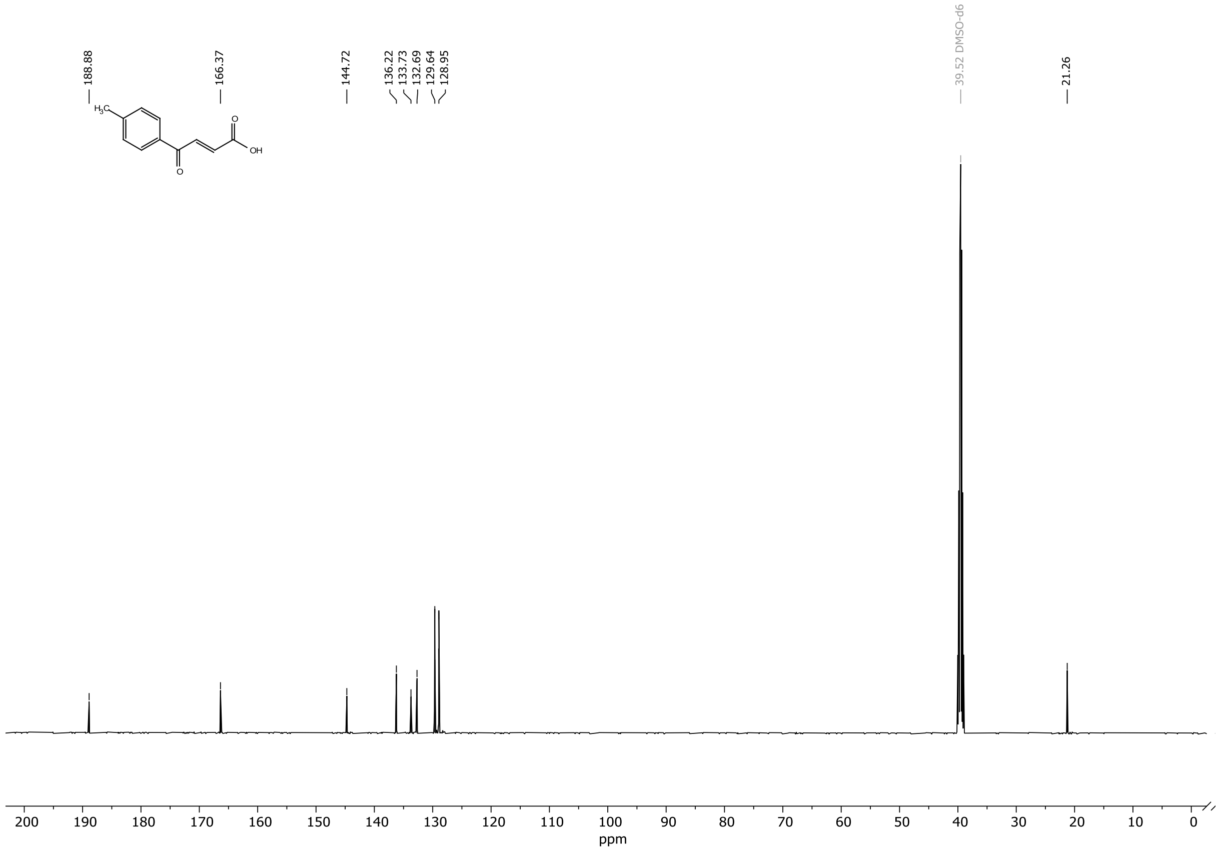

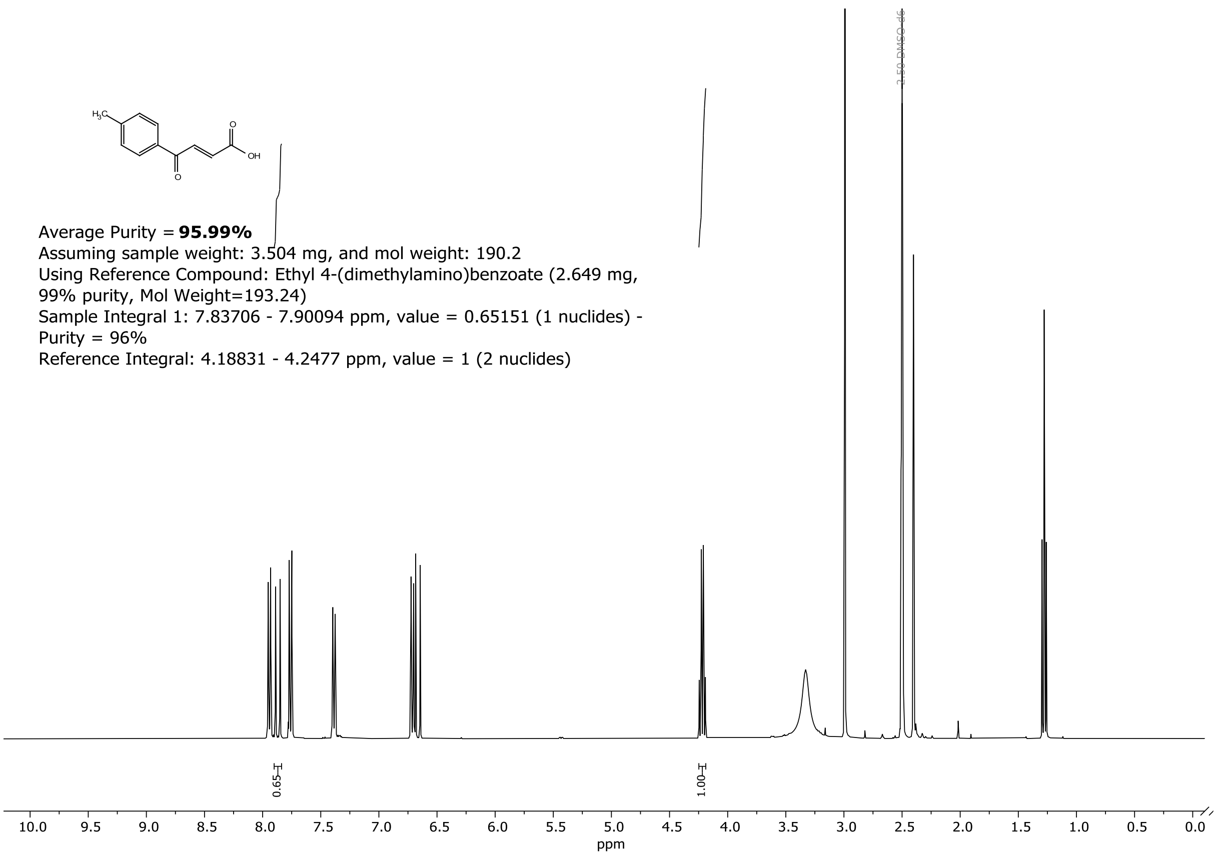
H3C
O
OH
Average Purity = **95.99%**
Assuming sample weight: 3.504 mg, and mol weight: 190.2
Using Reference Compound: Ethyl 4-(dimethylamino)benzoate (2.649 mg, 99% purity, Mol Weight=193.24)
Sample Integral 1: 7.83706 - 7.90094 ppm, value = 0.65151 (1 nuclides) - Purity = 96%
Reference Integral: 4.18831 - 4.2477 ppm, value = 1 (2 nuclides)
2.50 DMSO-d6
0.65
1.00
10.0 9.5 9.0 8.5 8.0 7.5 7.0 6.5 6.0 5.5 5.0 4.5 4.0 3.5 3.0 2.5 2.0 1.5 1.0 0.5 0.0
ppm

Compound **4.5**

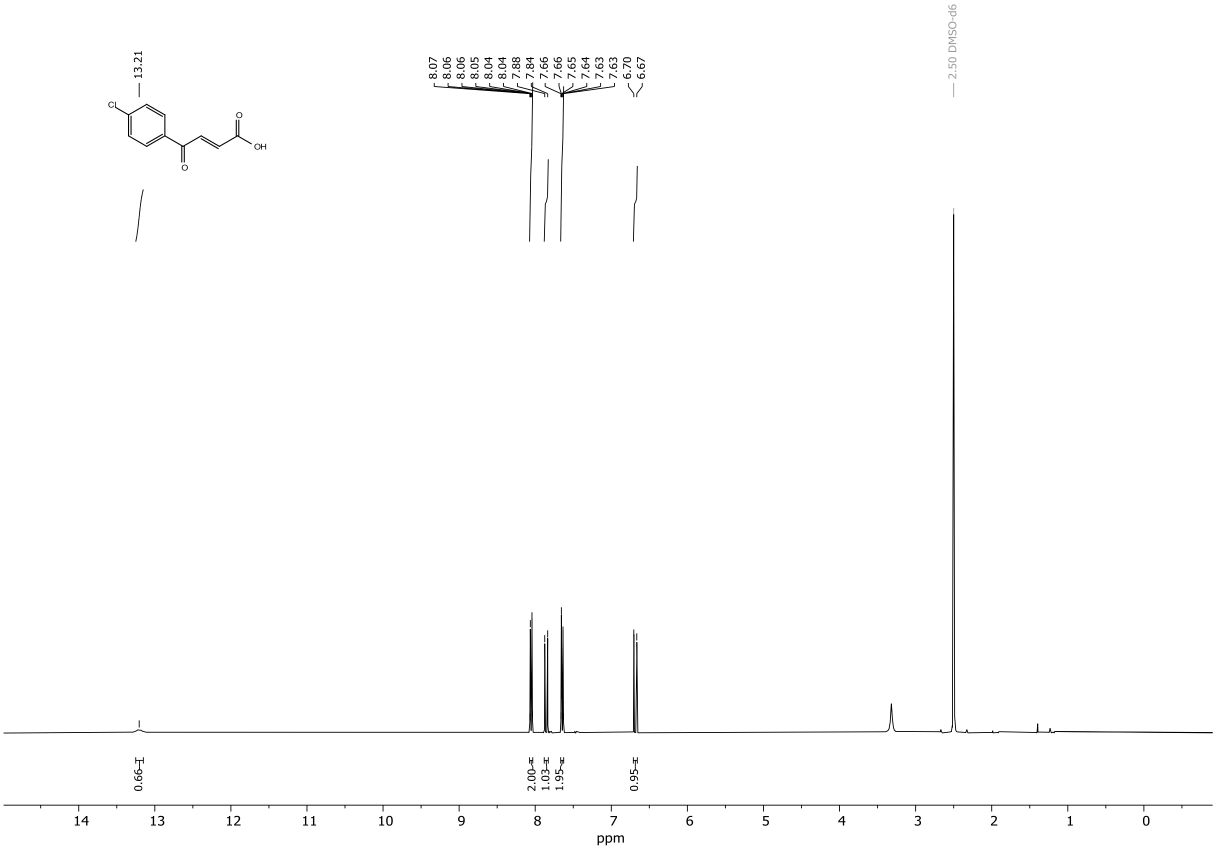
13.21
8.07
8.06
8.06
8.05
8.04
8.04
7.88
7.84
7.66
7.66
7.65
7.64
7.63
7.63
6.70
6.67
2.50 DMSO-d6
Cl
O
OH
O
0.66
2.00
1.03
1.95
0.95
14 13 12 11 10 9 8 7 6 5 4 3 2 1 0
ppm

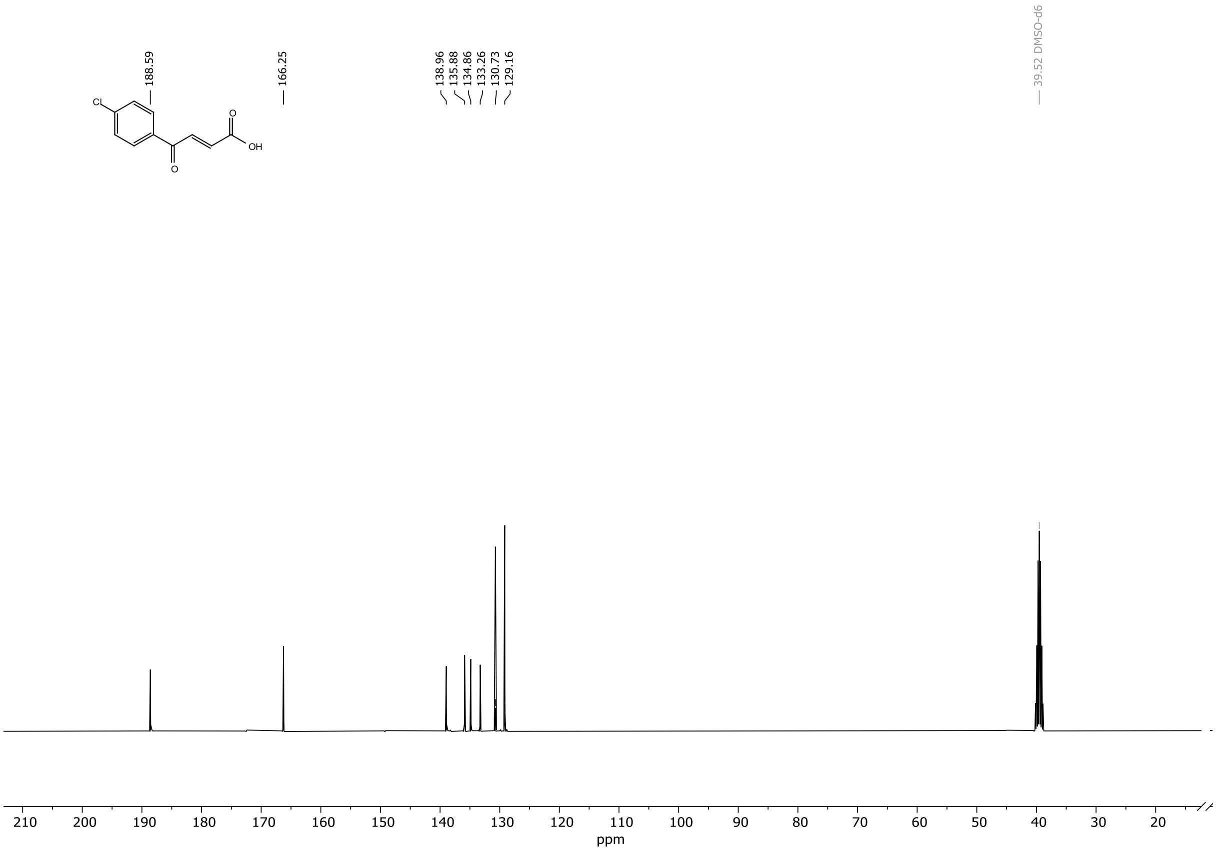
188.59
166.25
138.96
135.88
134.86
133.26
130.73
129.16
39.52 DMSO-d6
Cl
O
OH
O
210 200 190 180 170 160 150 140 130 120 110 100 90 80 70 60 50 40 30 20
ppm

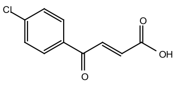

Average Purity = **98.92%**
Assuming sample weight: 4.367 mg, and mol weight: 210.6
Using Reference Compound: Ethyl 4-(dimethylamino)benzoate (3.625 mg, 99% purity, Mol Weight=193.24)
Sample Integral 1: 7.73007 - 7.78366 ppm, value = 1.10445 (1 nuclides)
- Purity = 98.9%
Reference Integral: 4.19095 - 4.24606 ppm, value = 1 (1 nuclides)

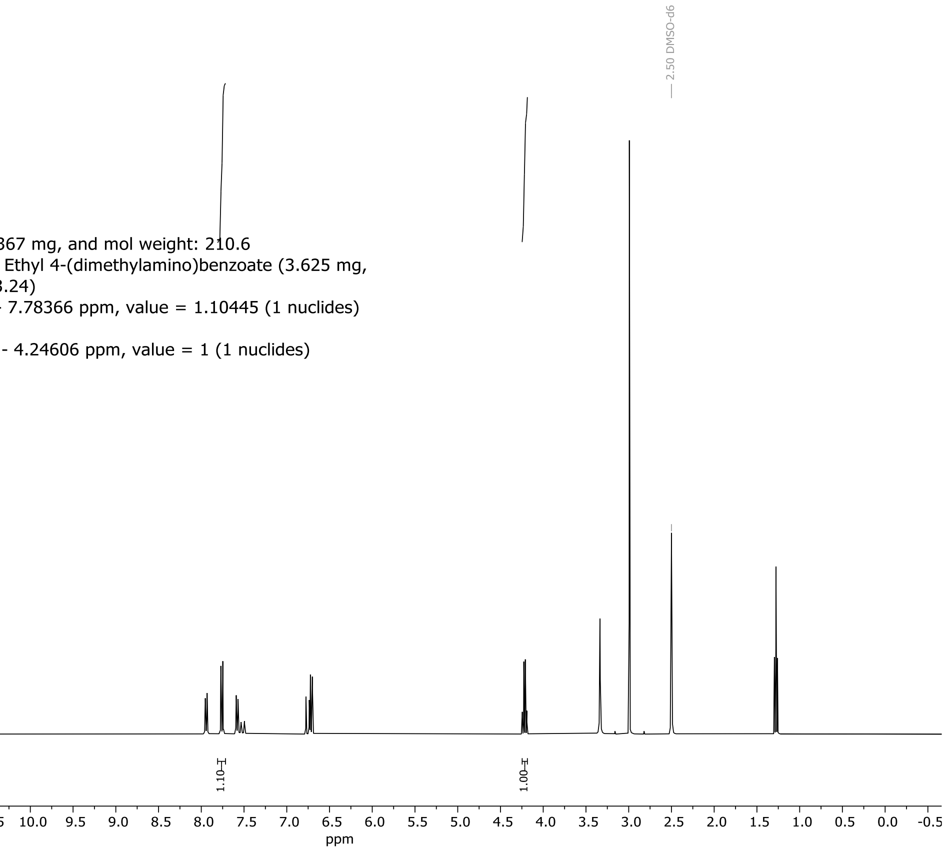

Compound **4.6**

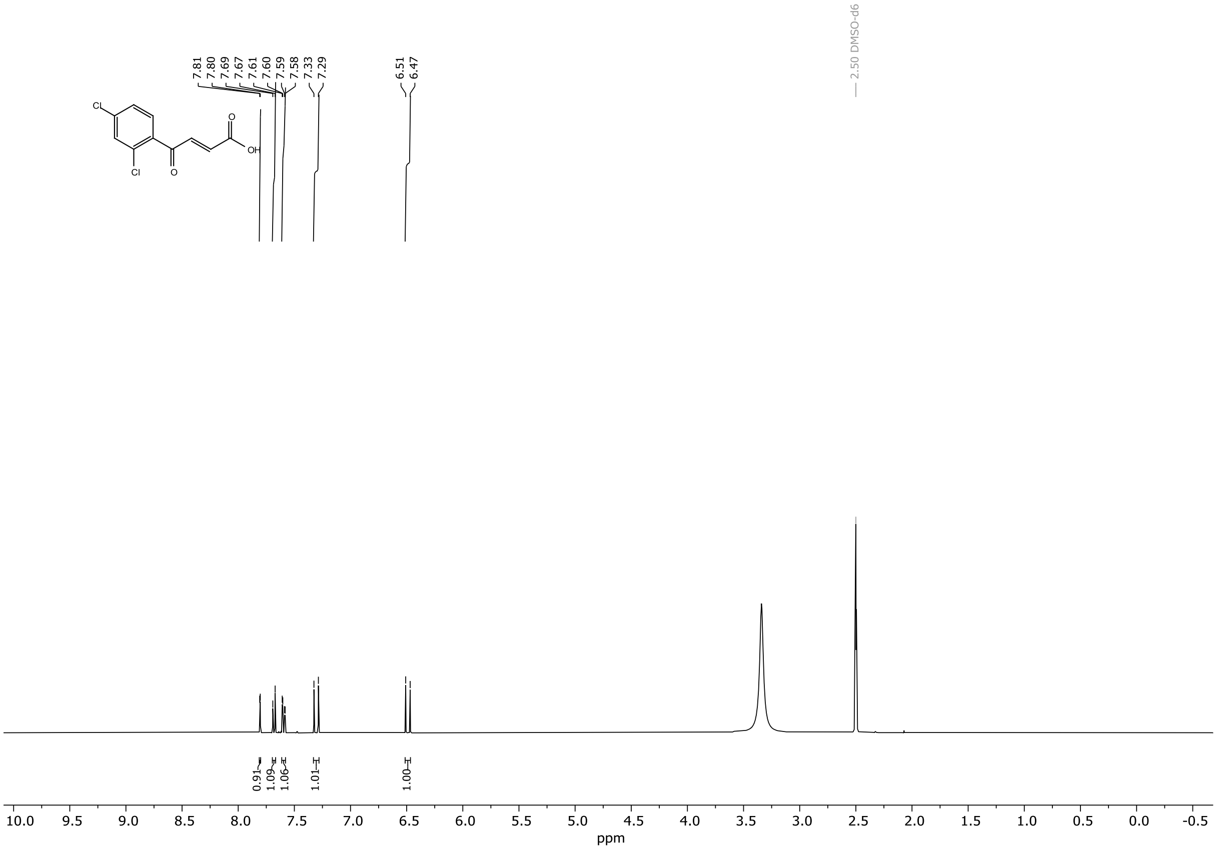
7.81
7.80
7.69
7.67
7.61
7.60
7.59
7.58
7.33
7.29
6.51
6.47
2.50 DMSO-d6
Cl
Cl
O
O
OH
0.91
1.09
1.06
1.01
1.00
10.0 9.5 9.0 8.5 8.0 7.5 7.0 6.5 6.0 5.5 5.0 4.5 4.0 3.5 3.0 2.5 2.0 1.5 1.0 0.5 0.0 -0.5
ppm

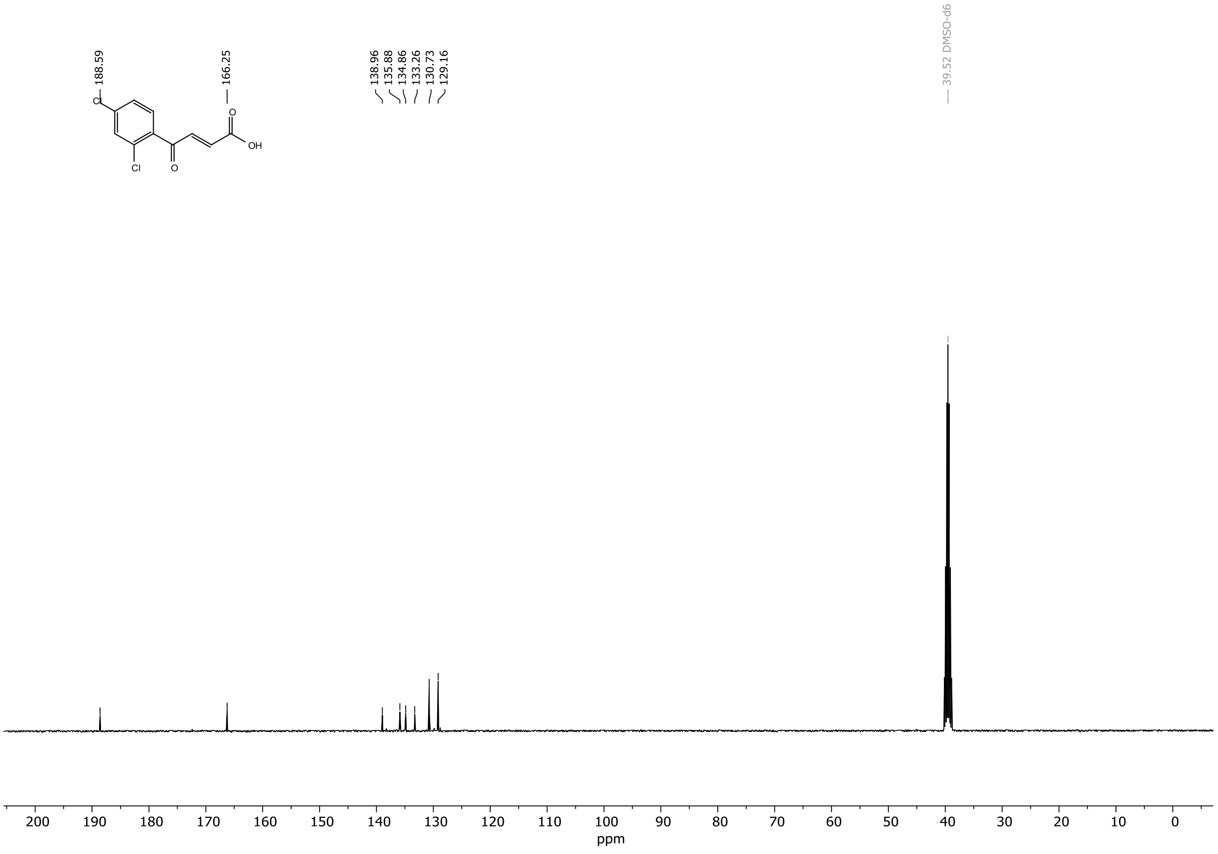
188.59
166.25
138.96
135.88
134.86
133.26
130.73
129.16
39.52 DMSO-d6
Cl
Cl
O
O
OH
200 190 180 170 160 150 140 130 120 110 100 90 80 70 60 50 40 30 20 10 0
ppm

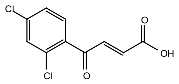

Average Purity = **95.53%**
Assuming sample weight: 1.751 mg, and mol weight: 245.06
Using Reference Compound: Ethyl 4-(dimethylamino)benzoate (3.013 mg, 99% purity, Mol Weight=193.24)
Sample Integral 1: 7.2687 - 7.35022 ppm, value = 0.2211 (1 nuclides) - Purity = 95.5%
Reference Integral: 4.19771 - 4.25755 ppm, value = 1 (2 nuclides)

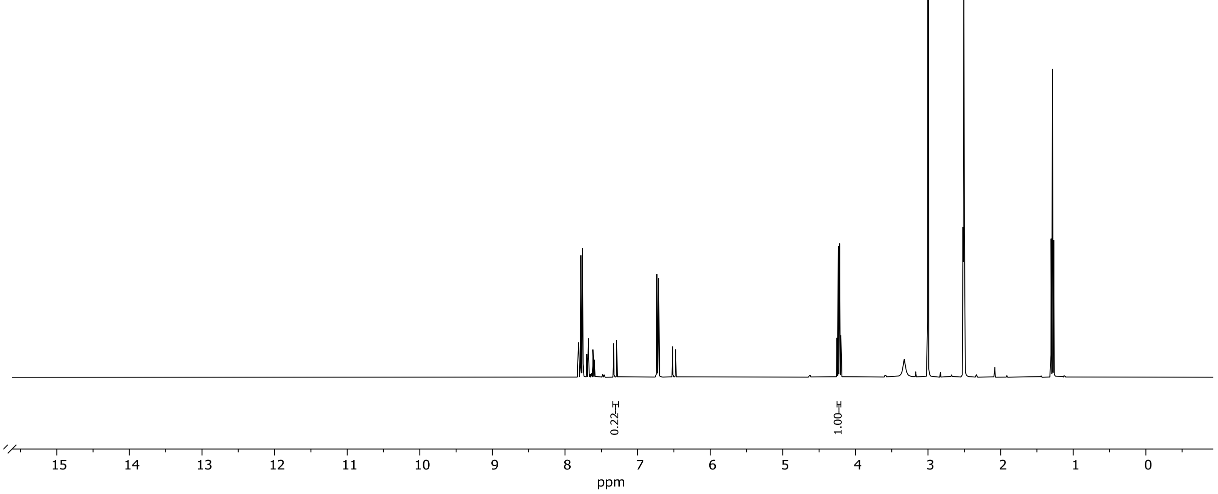

Compound **4.7**

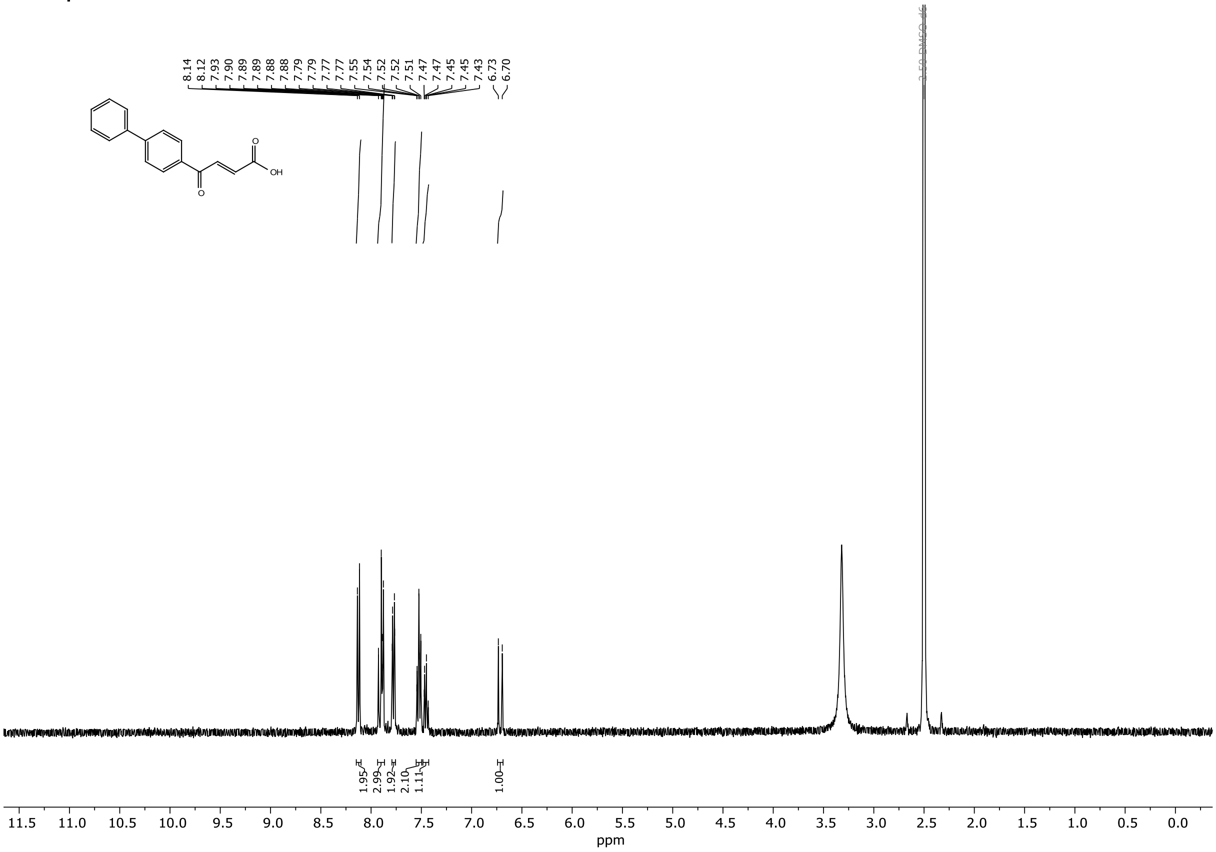

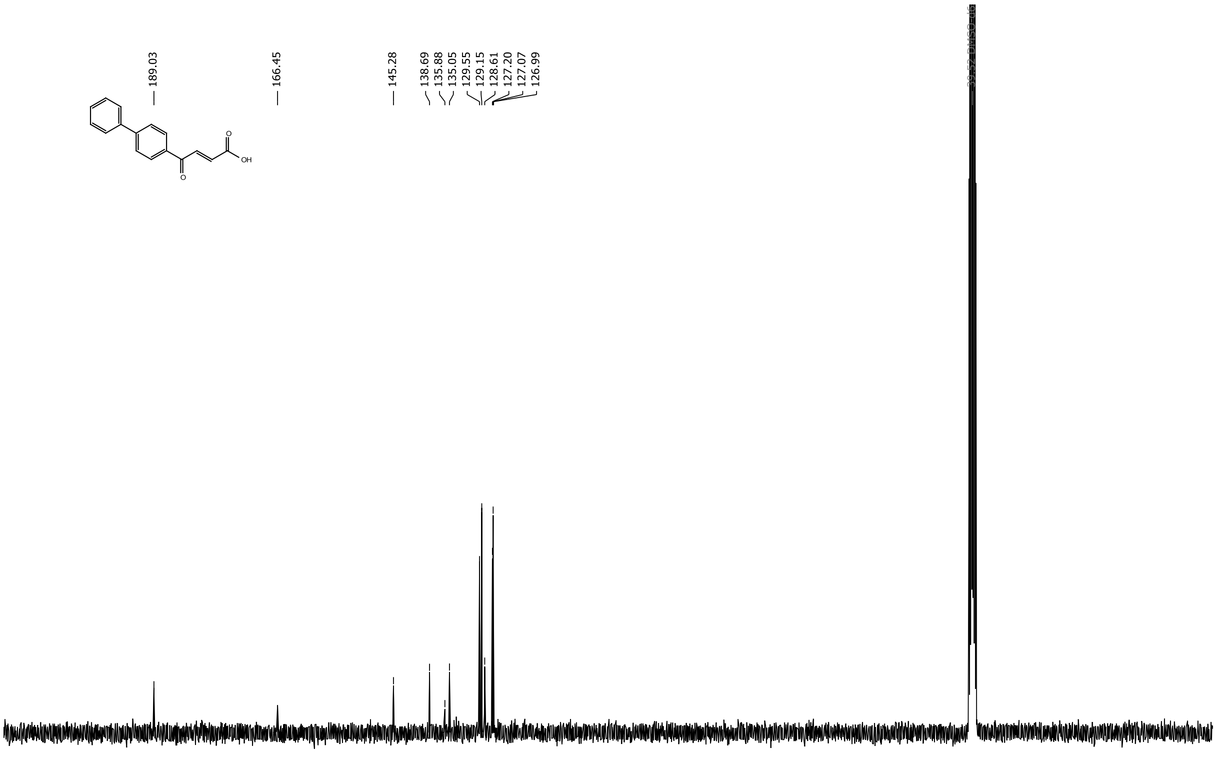

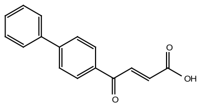

Average Purity = **97.16%**
Assuming sample weight: 1.384 mg, and mol weight: 252.3
Using Reference Compound: Ethyl 4-(dimethylamino)benzoate (1.813 mg, 99% purity, Mol Weight=193.24)
Sample Integral 1: 8.08961 - 8.17828 ppm, value = 0.5738 (1 nuclides) - Purity = 97.2%
Reference Integral: 4.19755 - 4.25667 ppm, value = 1 (1 nuclides)

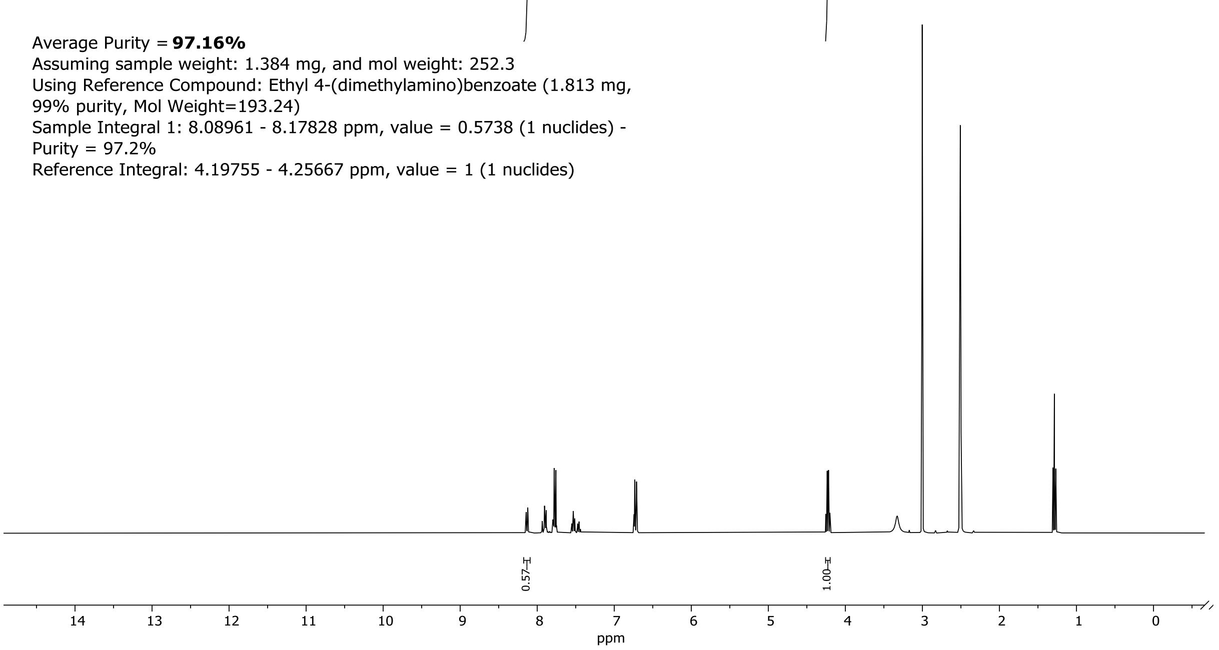

Compound **4.8**

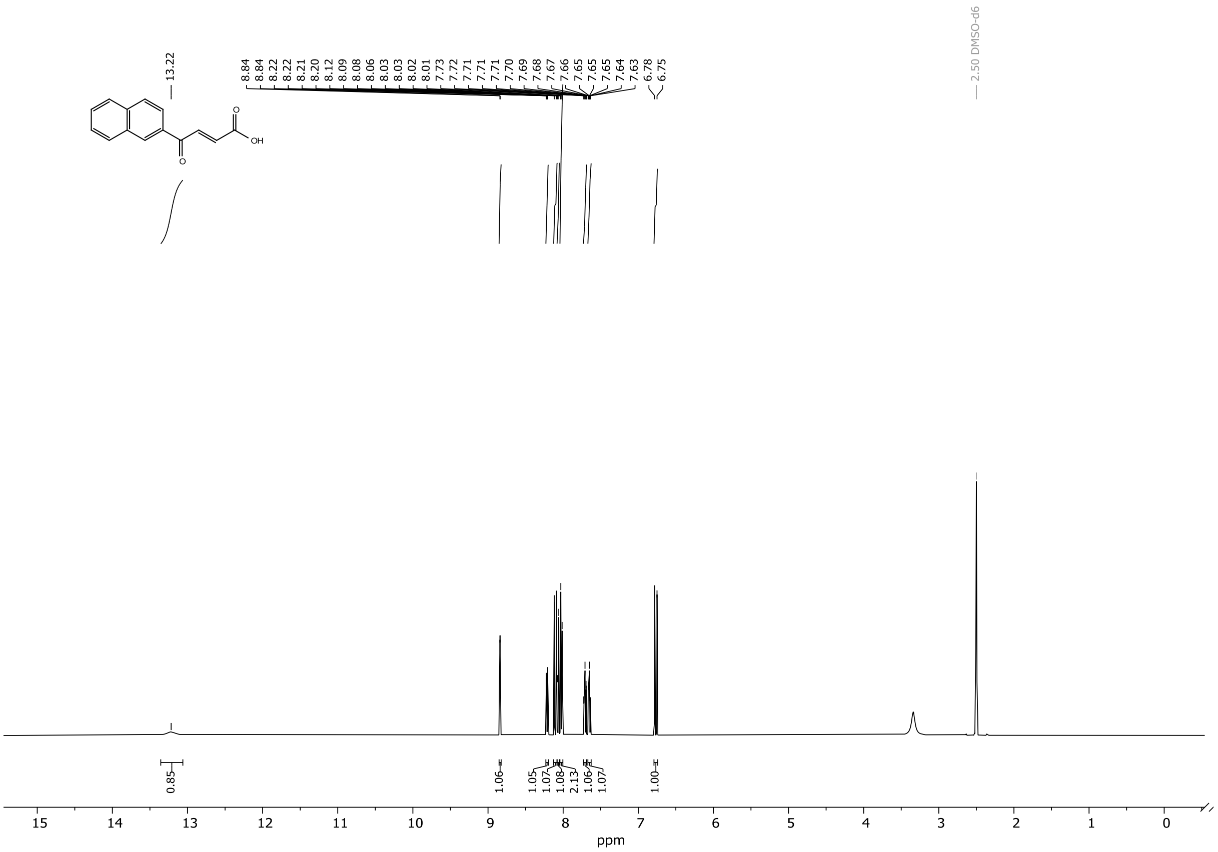
13.22
8.84
8.84
8.22
8.22
8.21
8.20
8.12
8.09
8.08
8.06
8.03
8.03
8.02
8.01
7.73
7.72
7.71
7.71
7.71
7.70
7.69
7.68
7.67
7.66
7.65
7.65
7.64
7.63
6.78
6.75
2.50 DMSO-d6
OH
O
0.85
1.06
1.05
1.07
1.08
2.13
1.06
1.07
1.00
15 14 13 12 11 10 9 8 7 6 5 4 3 2 1 0
ppm

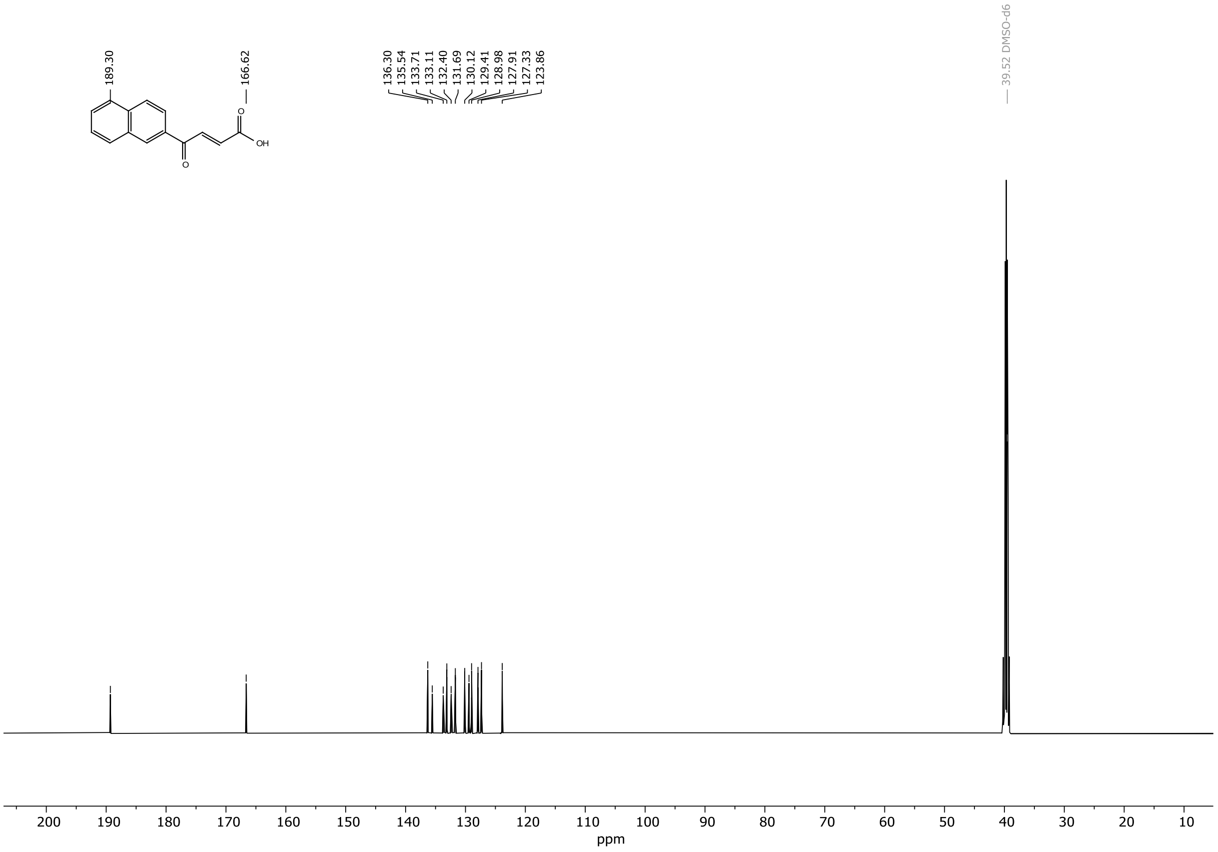
189.30
166.62
136.30
135.54
133.71
133.11
132.40
131.69
130.12
129.41
128.98
127.91
127.33
123.86
39.52 DMSO-d6
OH
O
200 190 180 170 160 150 140 130 120 110 100 90 80 70 60 50 40 30 20 10
ppm

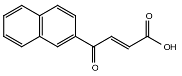

Average Purity = **98.87%**
Assuming sample weight: 1.784 mg, and mol weight: 226.3
Using Reference Compound: Ethyl 4-(dimethylamino)benzoate (2.718 mg, 99% purity, Mol Weight=193.24)
Sample Integral 1: 7.70554 - 7.74884 ppm, value = 0.27988 (1 nuclides) - Purity = 98.9%
Reference Integral: 3.68978 - 3.76015 ppm, value = 1 (2 nuclides)

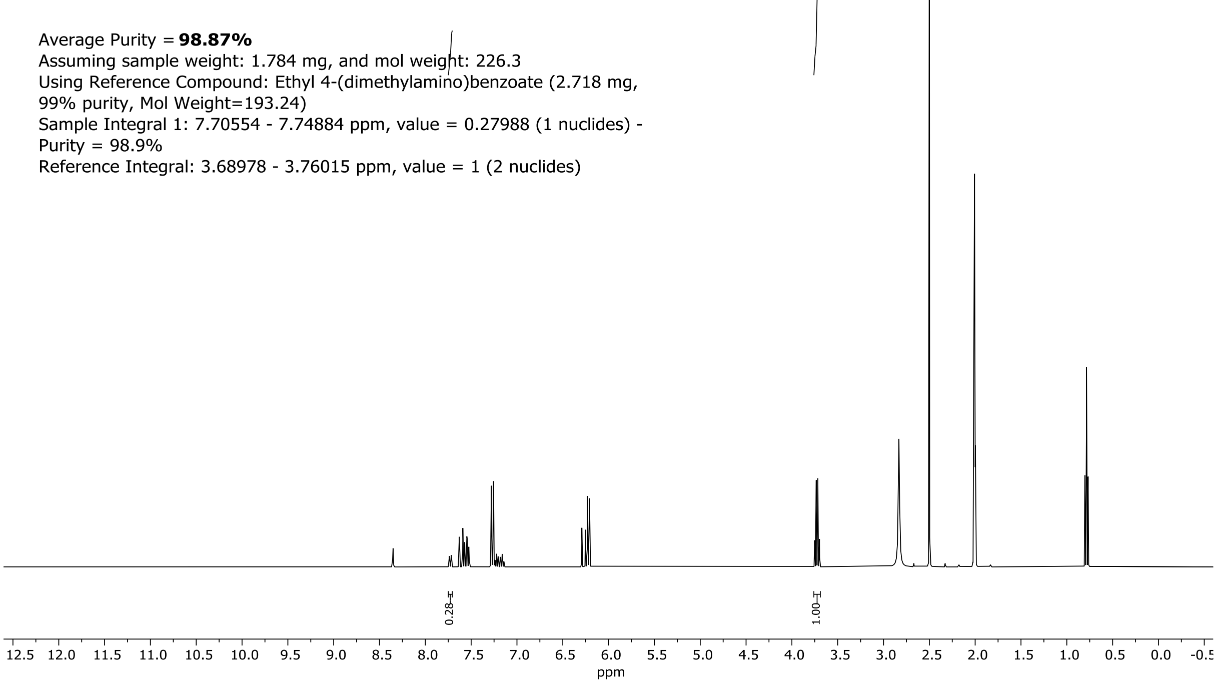

Supplement: MD-016-D5MD00531K-s001 [file MD-016-D5MD00531K-s001.pdf]
